# FSH and ApoE4 contribute to Alzheimer’s disease-like pathogenesis via C/EBPβ/δ-secretase in female mice

**DOI:** 10.1038/s41467-023-42282-7

**Published:** 2023-10-18

**Authors:** Jing Xiong, Seong Su Kang, Mengmeng Wang, Zhihao Wang, Yiyuan Xia, Jianming Liao, Xia Liu, Shan-Ping Yu, Zhaohui Zhang, Vitaly Ryu, Tony Yuen, Mone Zaidi, Keqiang Ye

**Affiliations:** 1 Department of Pathology and Laboratory Medicine, Emory University School of Medicine, Atlanta, GA 30322 USA; 2 Department of Neurology, Renmin Hospital of Wuhan University, Wuhan, 430060 Hubei Province China; 3 Faculty of Life and Health Sciences, Shenzhen Institutes of Advanced Technology, Chinese Academy of Sciences, Shenzhen, Guangdong 518055 China; 4 Department of Neurosurgery, Renmin Hospital of Wuhan University, Wuhan, 430060 Hubei Province China; 5 Department of Anesthesiology, Emory University School of Medicine, Atlanta, GA 30322 USA; 6 Mount Sinai Bone Program, Department of Medicine and Department of Orthopedics, Mount Sinai School of Medicine, New York, NY 10029 USA

**Keywords:** Alzheimer's disease, Menopause

## Abstract

Alzheimer’s disease (AD) is the most common dementia. It is known that women with one ApoE4 allele display greater risk and earlier onset of AD compared with men. In mice, we previously showed that follicle–stimulating hormone (FSH), a gonadotropin that rises in post–menopausal females, activates its receptor FSHR in the hippocampus, to drive AD–like pathology and cognitive impairment. Here we show in mice that ApoE4 and FSH jointly trigger AD-like pathogenesis by activating C/EBPβ/δ-secretase signaling. ApoE4 and FSH additively activate C/EBPβ/δ-secretase pathway that mediates APP and Tau proteolytic fragmentation, stimulating Aβ and neurofibrillary tangles. Ovariectomy-provoked AD-like pathologies and cognitive defects in female ApoE4-TR mice are ameliorated by anti-FSH antibody treatment. FSH administration facilitates AD-like pathologies in both young male and female ApoE4-TR mice. Furthermore, FSH stimulates AD-like pathologies and cognitive defects in ApoE4-TR mice, but not ApoE3-TR mice. Our findings suggest that in mice, augmented FSH in females with ApoE4 but not ApoE3 genotype increases vulnerability to AD-like process by activating C/EBPβ/δ-secretase signalling.

## Abstract (web summary)

Follicle stimulating hormone (FSH) has been shown to Alzheimer’s disease like pathology in rodent models. Here the authors show using cellular and animal models that ApoE4 and FSH collectively act to trigger AD-like pathogenesis, by activating C/EBPβ/δ-secretase signalling.

## Introduction

Alzheimer’s disease (AD) is a progressive neurodegenerative disease featured by dysfunctions in memory, attention, language, and daily living activities. The pathological hallmarks include the extracellular senile plaques, primarily composed of Aβ polypeptides, and intra-neuronal neurofibrillary tangles (NFT), mainly consisting of hyper-phosphorylated and truncated Tau. The AD brain displays extensive neurodegeneration, associated with chronic inflammation. The female prevalence of AD is well documented and approximately 2/3 of AD patients are women. Epidemiological studies indicate a sex-specific association between APOE ε4 allele and female sex. Women with one cope of APOE ε4 allele display a four-fold increased risk of AD when compared to women homozygous for APOE ε3 allele, whereas women with two copies of APOE ε4 allele exhibit a 15-fold increase in risk. And women ε3/ε4 allele carriers often show early-onset, faster age-related decline and greater deterioration of cognition than ε3/ε4 men carriers^1^. A recent study has shown that Tau pathology may contribute to the increased risk of AD in women carrying one cope of APOE ε4 allele compared to men^2^.

FSH (follicle-stimulating hormone), a gonadotropin secreted by the gonadotropic cells of the anterior pituitary gland, is a central regulator of male and female reproductive function. FSH is a hetero-dimeric pituitary glycoprotein consisting of an α-subunit, which is common to other glycoprotein hormones, and a specific β-subunit. FSH binds and activates the FSH receptor (FSHR), which belongs to the 7 transmembrane G protein-coupled receptor (GPCR) family^3^. The transduction of the hormone-induced signal is mediated by the FSH specific FSHR, of which the action relies on the interaction with a number of intracellular effectors. The FSHR is primarily expressed in Sertoli cells in testis and granulosa cells in ovaries^4^. Knockout of the FSH β-subunit or the FSHR genes in mice results in significant reproductive defects in both sexes^5,6^. FSH has a primary function in procreation, wherein it induces estrogen production in females and regulates spermatogenesis in males^7^. The early menopausal transition is associated with a sharp rise in serum FSH levels, even when serum estrogen levels remain within normal limits^8^. Serum FSH level starts to rise about 2–3 years before menopause when serum estrogen levels were normal. Interestingly, FSH regulates bone mass via increasing bone resorption by osteoclasts^9^ and regulates body fat^10^. Blocking FSH action on its binding site to receptor not only increases bone mass^11^ but also reduces body fat^10^. These data provide a new perspective, FSH may be an important hormone in peri-menopause, which exhibits alternative mechanisms in parallel with the role of estrogen. Ovarian failure during late peri-menopause is associated with a sharp rise in serum FSH, which coincides with the most rapid rate of bone loss and the onset of visceral adiposity^12^. It is noteworthy that the epidemiology study demonstrates that surgical menopause is linked to cognitive decline and AD pathology in women who have undergone surgical menopause^13^. For instance, FSH levels are significantly higher in estrogen-free women with AD^14^. Earlier age at surgical menopause is associated with faster decline in global cognition, and also associated with increased AD neuropathology, in particular neuritic plaques^13^. There is an increased risk of cognitive impairment or dementia among women who undergo bilateral oophorectomy before menopause^15^. Regardless of whether a concurrent hysterectomy is performed, a unilateral oophorectomy increases the risk of cognitive decline or dementia^16,17^.

C/EBPβ, a transcription factor of the basic-leucine zipper (bZIP) class and a member of the CCAAT/enhancer binding protein (C/EBP) family, is implicated in a number of biological processes, including cell energy consumption, cell proliferation, and cell differentiation^18,19^. C/EBPβ has a crucial function in inflammation^20^. Numerous pro-inflammatory genes contain potential consensus sequences of C/EBPβ^21^, and macrophages^22^ and glial cells^23^ exhibit an increase in C/EBPβ levels under the exposure to pro-inflammatory stimuli. In C/EBPβ-null brain, both pro-inflammatory genes and the neurotoxic consequences of activated microglia are diminished^24^. It is interesting to note that neuroprotection following ischemic^25^ or excitotoxic injuries^26^ is provided by C/EBPβ deficiency. Remarkably, C/EBPs are increased in the brain of AD patients^27,28^, and Aβ induces C/EBPβ and δ activation in glia cells^29^. We recently discovered that age-dependent increase in C/EBPβ occur both in the human and mice brains^30^. C/EBPβ functions as a crucial transcription factor of δ-secretase, which is also named AEP (asparagine endopeptidase, gene name: *LMGN*). Increased levels of C/EBPβ contribute to the development of AD pathologies by partially enhancing AEP expression^30^, which acts as δ-secretase and then cleaves both APP and Tau at N585 and N368 residues, respectively, resulting Aβ production and Tau aggregation. Deletion of δ-secretase significantly improves senile plaques and NFT (neurofibrillary Tangles) pathologies, and reverses cognitive impairments both in 5xFAD or Tau P301S mice^31,32^. Moreover, we found that C/EBPβ/δ-secretase pathway is spatiotemporally activated and mediates AD pathologies^33^. Notably, C/EBPβ mediates steroidogenic acute regulatory protein (StAR) and prostaglandin endoperoxide synthase 2 genes in ovarian granulosa cells upon FSH stimulation^34–36^. On the other hand, FSH induces various C/EBP isoforms and activates C/EBPβ transcriptional activities via cAMP in Sertoli cells^37^. Women ε3/ε4 allele carriers exhibit faster decline of cognition than elderly ε3/ε4 men in younger age^1^, and FSH is significantly augmented in estrogen-free women with AD^14,38^. Most recently, we report that FSH activates its FSHR in the brain, driving women more susceptible for AD pathogenesis, and blockade of FSH improves cognition in mice with AD^39^. Hence, in the current work, we test the hypothesis that ApoE4 and FSH may additively predispose females to AD pathogenesis via activating C/EBPβ/δ-secretase signaling pathway.

## Results

### FSH and ApoE4 additively activate C/EBPβ/δ-secretase in primary neurons

**Fig. 1:**
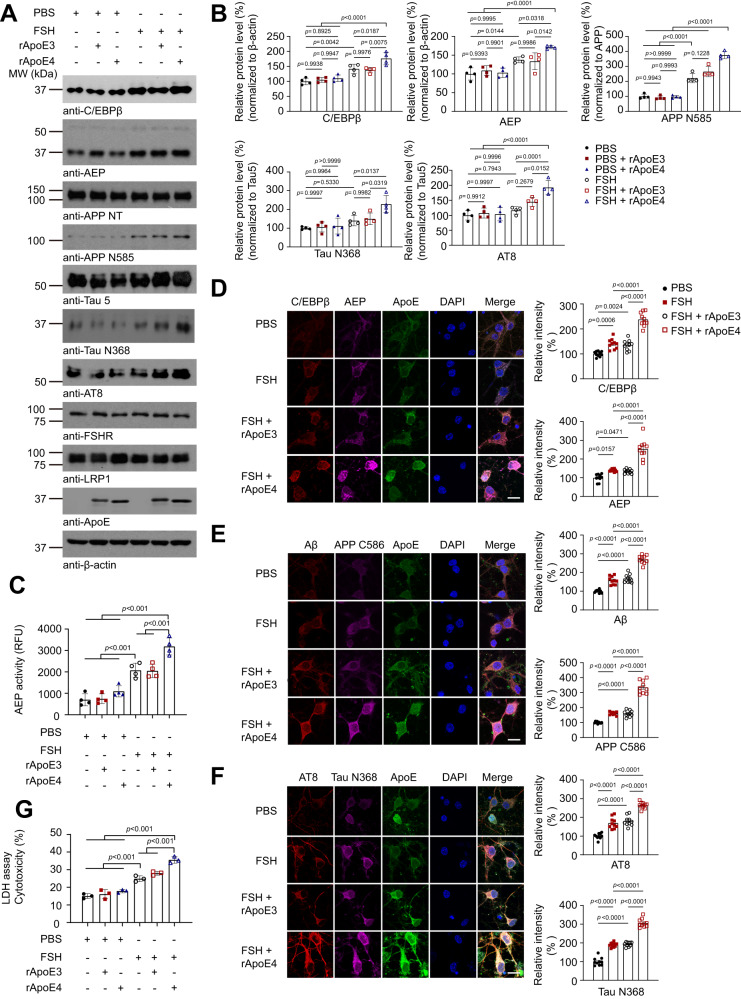
FSH and ApoE4 additively activate C/EBPβ/δ-secretase pathway. Primary rat neurons (DIV. 13) were treated with vehicle, recombinant human FSH (30 ng/ml), combined with human recombinant ApoE3 or ApoE4 proteins (100 nM) for 48 hours. Then the cells were harvested for western blot (**A–B**), AEP enzymatic assay (**C**), immunofluorescent staining (**D**–**F**), and LDH cytotoxicity assay (**G**). **A** Represent image of four independent experiments. **B** Statistical analysis of protein expression. Data are shown as mean ± SEM. (*n* = 4 independent experiments, one-way ANOVA followed by Tukey’s multiple comparisons test). **C** AEP enzymatic activity was assessed. Data represent mean ± SEM of four independent experiments (one-way ANOVA test, followed by Tukey’s multiple comparisons test). **D**–**F** Immunofluorescent staining showed the impact of FSH (30 ng/ml) on C/EBPβ (red)/AEP (green) (**D**), Aβ (red)/APP C586 (green) (**E**), AT8 (red)/Tau N368 (green) (**F**) in rat cortical neuron cultures with rApoE3 or rApoE4 proteins (Scale bar, 10 μm). Data represent mean ± SEM (*n* = 10 slices from three independent experiments, one-way ANOVA test followed by Tukey’s multiple comparisons test for C/EBPβ, AEP, APP C586, AT8, and Tau N368 quantification, Brown-Forsythe and Welch ANOVA tests for Aβ quantification). **G** LDH cytotoxicity assay showed FSH combined with rApoE4 treatment increased the cytotoxicity significantly. Data represent mean ± SEM of three independent experiments (one-way ANOVA test followed by Tukey’s multiple comparisons test).

We have recently reported that FSHR is expressed in human brain, especially in neurons^39^. More FSH and FSHR were co-localized in female AD brains than controls. FSHR were expressed in human and mouse brains and rat primary cortical neurons. FSH activates C/EBPβ/δ-secretase pathway and stimulates AD pathogenesis in female 3xTg mice^39^. To investigate whether ApoE4 is implicated in activating C/EBPβ/δ-secretase pathway by FSH, we used recombinant human FSH (30 ng/ml) to stimulate primary neuronal cultures in the presence of rApoE3 or rApoE4 recombinant proteins. Compared to vehicle control, FSH robustly increased C/EBPβ and active AEP, which subsequently cleaved APP and Tau into APP N585 and Tau N368 truncates respectively. Tau N368 fragmentation was tightly associated with prominent Tau phosphorylation AT8. By contrast, FSHR and LRP1, an ApoE receptor, remained constant (Fig. 1A, B). Quantitative enzymatic assay showed that FSH significantly increased AEP protease activities as compared with PBS, and rApoE4 but not rApoE3 further augmented FSH stimulatory effect (Fig. 1C). Immunofluorescent (IF) staining validated immunobloting (IB) observations that FSH activated C/EBPβ/AEP signaling, resulting in APP and Tau proteolytic fragmentation, associated with both Aβ and AT8 escalation. Remarkably, ApoE4 additively increased FSH’s actions (Fig. 1D–F). In alignment with these findings, LDH assay revealed that FSH significantly elevated neurotoxicity as compared to PBS, which was further augmented by ApoE4 but not ApoE3 (Fig. 1G). Therefore, ApoE4 and FSH jointly activate C/EBPβ/δ-secretase pathway and trigger both APP and Tau proteolytic cleavage, leading to Aβ and p-Tau augmentation and neuronal cell death.

Moreover, ApoE is mainly produced by Astrocyte in the brain. Astrocyte secreted lots of lipidated ApoE to maintain brain hemostasis. In order to examine the effects of lipidated ApoE on the C/EBPβ/δ-secretase pathway and the interaction with FSH, we used HEK293T cells to produce lipidated ApoE3 and ApoE4 and use lipidated ApoE3 or ApoE4 combined with hFSH (30 ng/ml) to stimulate the primary neuronal cultures. We found FSH mixed with lipidated ApoE4 robustly increased C/EBPβ and active AEP, which subsequently cleaved APP and Tau into APP N585 and Tau N368 truncates, respectively (Supplementary Fig. 1A, B). Lipidated ApoE4 significantly stimulated FSH to increase AEP protease activities as compared with rApoE3 and PBS (Supplementary Fig. 1C). In alignment with these findings, LDH assay revealed that FSH substantially elevated neural cell death, which was further augmented by lipidated ApoE4 but not lipidated ApoE3 (Supplementary Fig. 1D). Immunofluorescent (IF) staining confirmed that lipidated ApoE4 synergistically activated C/EBPβ/AEP signaling with FSH, resulting in APP and Tau proteolytic fragmentation, associated with both Aβ and AT8 escalation (Supplementary Fig. 1E–G). The lipidated ApoE proteins were validated on a NativePAGE gel and SDS-PAGE (Supplementary Fig 2). In summary, lipidated ApoE4 exhibited the similar effect to rApoE4 on C/EBPβ/δ-secretase pathway activation by FSH.

To further explore the joint effect by FSH and ApoE4, we employed human primary neurons, induced from ApoE3/E3 or ApoE4/E4 iPSC cells. After induction into mature neurons, we treated E3/3 or E4/4 primary neurons with different doses of FSH, followed by immunoblotting analysis. FSH dose-dependently activated C/EBPβ/δ-secretase pathway in ApoE3/3 primary neurons, leading to APP N585 and Tau N368 cleavage and AT8 augmentation. Remarkably, these events were evidently enhanced in ApoE4/4 neurons. We made the similar observations in two different sets of human iPSC-derived neurons (Supplementary Fig. 3A). The induced mature neurons were validated with neuronal biomarkers Tuj-1 and MAP2 co-staining (Supplementary Fig. 3B). Quantitative enzymatic assay showed that FSH progressively escalated AEP enzymatic activities in E3/3 neurons; whereas AEP activities were substantially higher in ApoE4/4 neurons, which were further elevated by FSH in a concentration-dependent manner (Supplementary Fig. 3C). Accordingly, FSH-stimulated Aβ40 and Aβ42 from human neurons exhibited the similar pattern, oscillated with the upstream effector AEP activities (Supplementary Fig. 3D). Hence, FSH stimulates C/EBPβ/AEP signaling in human neurons and ApoE4 exacerbates FSH stimulatory effects.

### FSH administration accelerates AD pathologies and cognitive deficits in young male and female ApoE4-TR mice

**Fig. 2:**
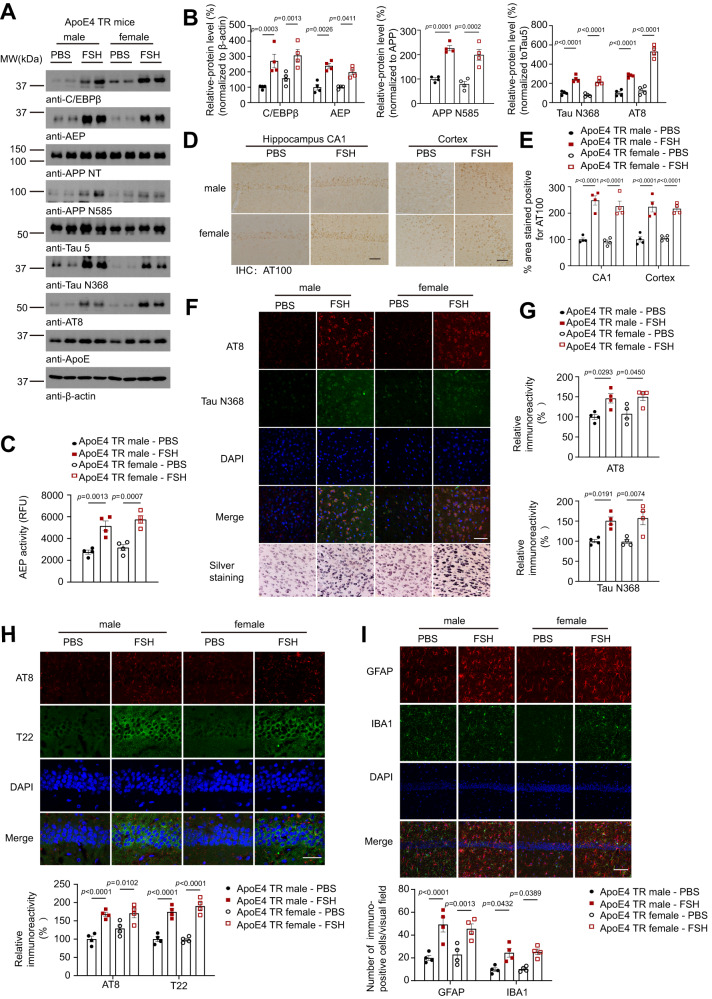
FSH triggers C/EBPβ/δ-secretase signaling and AD pathology in both male and female ApoE4-TR mice. Four months old male and female ApoE4-TR mice were treated with FSH (5 IU per day, 6 days a week) by intraperitoneal injection for 3 months. **A** Representative image of western blot showed increased C/EBPβ, AEP, cleaved APP, Tau, and p-Tau expression in male and female ApoE4-TR mouse brains after FSH administration. **B** Quantification of western blot data. Data are shown as mean ± SEM. (*n* = 4 mice per group, two-way ANOVA for C/EBPβ, AEP, Tau N368, AT8, one-way ANOVA followed by Tukey’s multiple comparisons test for APP N585). **C** AEP enzymatic activities assay. Data are shown as mean $\pm$ SEM (*n* = 4 mice per group, one-way ANOVA followed by Tukey’s multiple comparisons test). **D**, **E** IHC staining showed AT100 immuno-reactivity in the hippocampus and the cortex (scale bar, 50 μm). Data are presented as mean $\pm$ SEM (*n* = 4 mice per group, two-way ANOVA). **F** Immunofluorescence staining showed AT8 (red) and Tau N368 (green) immunoreactivity in the cortex of male and female ApoE4 TR mice after FSH treatment (scale bar, 50 μm). Representative image of Silver staining in the cortical regions (the lower panels). **G** Quantification of AT8 and Tau N368 immuno-reactivity. Data are presented as mean $\pm$ SEM (*n* = 4 mice per group, one-way ANOVA followed by Tukey’s multiple comparisons test). **H**, **I** IF staining of AT8 (red) and T22 (green) (**H**), GFAP (red) and IBA1 (green) (**I**) in the hippocampus. (scale bar = 50 μm (**H**), 100 μm (**I**)). Data represent mean $\pm$ SEM (*n* = 4 mice per group, two-way ANOVA).

To explore whether FSH accelerates AD pathologies in ApoE4-TR mice, we intraperitoneally (i.p.) injected FSH (5 IU/d) to 4 months old female and male mice consecutively for 3 months. FSH strongly escalated C/EBPβ and its downstream target AEP expression in both male and female ApoE4-TR mouse brains as compared to PBS. Notably, FSH also robustly stimulated AEP activation. Consequently, APP N585, Tau N368 and p-Tau levels were prominently elevated (Fig. 2A, B). Quantitative analysis revealed AEP enzymatic activities were highly increased by FSH in both male and female brains (Fig. 2C). Immunohistochemistry (IHC) showed that p-Tau AT100 signals were increased by FSH in the hippocampus and cortex of both sexes (Fig. 2D, E). IF analysis revealed that p-Tau AT8/Tau N368 co-staining also exhibited the similar pattern in alignment with IHC findings. Silver staining validated intra-neuronal aggregated inclusions in the cortical regions with female stronger than male (Fig. 2F, G). Moreover, T22, a biomarker for aggregated Tau pathology, and AT8 co-staining also confirmed that FSH strongly enhanced p-Tau and its fibrillization in the hippocampus of both male and female mouse brains (Fig. 2H). GFAP and Iba-1 staining showed that FSH escalated the astrogliosis and microglia activation in the brain, respectively (Fig. 2I).

**Fig. 3:**
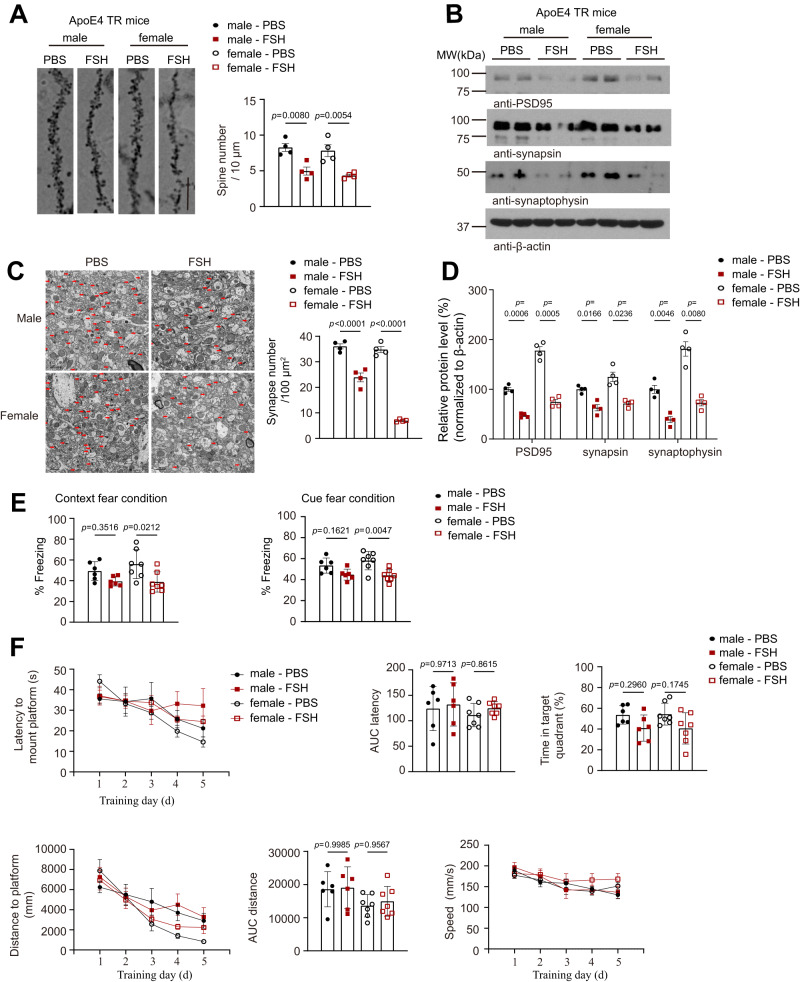
FSH triggers AD pathology and cognitive dysfunctions in both male and female ApoE4-TR mice. **A** A Golgi stain on brain sections from the CA1 region of the hippocampus revealed fewer spines in both male and female ApoE4 TR mice followed by FSH treatment. (scale bar, 10 μm). Quantification of the dendritic spine density, which was calculated as the number of dendritic branch per 10 μm dendrite. Data are shown as mean $\pm$ SEM (*n* = 4 mice per group, one-way ANOVA followed by Tukey’s multiple comparisons test). **B** Representative image of western blot showed FSH treatment decreased PSD95, synapsin and synaptophysin expression, triggering synapse loss. **C** The synapses were detected by electron microscopy (scale bar, 1 μm). Arrows indicated the synapses. Data are presented as mean $\pm$ SEM (*n* = 4 mice per group, one-way ANOVA followed by Tukey’s multiple comparisons test). **D** Statistical analysis of western blot data (mean ± SEM, *n* = 4 mice per group, two-way ANOVA). **E** Fear condition tests, including context (left) and cue (right) fear conditioning cognition. Data represent mean $\pm$ SEM (*n* = 6 (male group) or 7 (female group) mice per group, one-way ANOVA followed by Tukey’s multiple comparisons test). **F** Morris water maze analysis of cognitive functions. Data are shown as mean $\pm$ SEM. (*n* = 6 (male group) or seven (female group) mice per group, two-way ANOVA for Latency, Distance, and Speed analyze, one-way ANOVA for AUC latency, Time in the target quadrant, and AUC distance).

Golgi staining indicated that FSH significantly reduced dendritic spines in the hippocampal neurons from both sexes (Fig. 3A). In addition, both sexes showed a decrease in synaptic proteins expression (Fig. 3B, D). Therefore, these findings support that FSH treatment triggers synaptic degeneration in ApoE4-TR brains from both male and female mice. Moreover, VGLUT1 and GAD67 staining demonstrated that FSH treatment reduced excitatory neurons and inhibitory GABAergic interneurons in both sexes (Supplementary Fig. 4A, B). Consequently, electronic microscopy (EM) analysis showed that FSH robustly reduced synapses in both sexes (Fig. 3C). Fear conditioning assays showed that FSH considerably decreased both contextual and cued fear conditions in female ApoE4-TR mice not male mice (Fig. 3E), in line with the substantial Tau pathologies found in the cortex. In Morris Water Maze (MWM) behavioral tests, mice treated with FSH had a substantially longer latency to the submerged platform than mice given PBS controls.

These mice spent less time than the controls in the target quadrant during the probe trail test. However, the differences were not statistically significant (Fig. 3F). Hence, FSH administration elevates the AD pathogenesis in both male and female ApoE4-TR mice, however leading to contextual memory deficits in female mice.

### FSH induces AD pathologies and cognitive deficits in ApoE4-TR but not ApoE3-TR mice

**Fig. 4:**
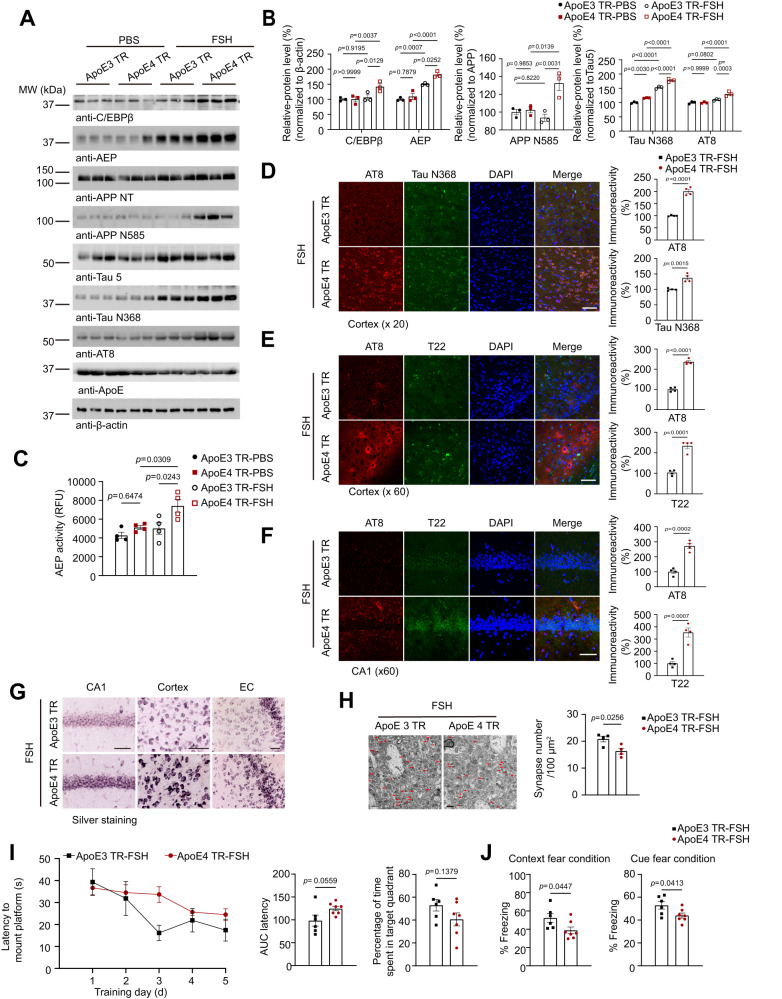
FSH administration accelerates AD pathology and cognitive deficits in ApoE4 but not ApoE3-TR mice. Four months old female ApoE3 TR and ApoE4 TR mice were treated with FSH (5 IU, per day, 6 days a week) by intraperitoneal injection for 3 months. **A** Representative image of western blot showing the expression of C/EBPβ, AEP, cleaved APP, Tau and p-Tau in the mice hippocampus. **B** Quantification of the protein expression, data are presented as mean ± SEM. (*n* = 3 mice per group, two-way ANOVA or one-way ANOVA followed by Tukey’s multiple comparisons test). **C** AEP enzymatic activity in the hippocampus. (*n* = 4 mice per group, one-way ANOVA followed by Tukey’s multiple comparisons test). **D**–**F** Co-staining of AT8 (red)/Tau N368 (green) (**D**), AT8 (red)/T22 (green) (**E**) in the cortex and AT8 (red)/T22 (green) in the hippocampus (**F**). (scale bar, 50 μm). The immunoreactivities of AT8, Tau N368 and T22 were quantified. Data are presented as mean $\pm$ SEM (*n* = 4 mice per group, unpaired *t* test with Welch’s correction). **G** Silver staining of the hippocampal CA1, and prefrontal cortex and entorhinal cortex (EC) regions (scale bar, 50 μm). **H** Electron microscopy analysis of the synapses (scale bar, 1 μm). Arrows indicated the synapses. Data are shown as mean $\pm$ SEM (*n* = 4 mice per group, unpaired *t* test with Welch’s correction). **I** Morris water maze analysis of cognitive functions. Data are shown as mean $\pm$ SEM (*n* = 6 mice per group in ApoE3 TR group, *n* = 7 mice per group in ApoE4 TR group, two-way ANOVA for Latency, unpaired *t* test with Welch’s correction for AUC latency, Time in target quadrant). **J** Fear condition tests. Context (left) and cue (right) fear condition cognition was impaired by FSH treatment in ApoE4-TR mice compared with ApoE3-TR mice. Data represent mean $\pm$ SEM (*n* = 6 mice per group in ApoE3 TR group, *n* = 7 mice per group in ApoE4 TR group, unpaired *t* test with Welch’s correction).

There’s no difference of the serum FSH level between ApoE3 and ApoE4 mice at different ages (Supplementary Fig. 5A). To investigate whether FSH preferentially stimulates AD pathologies in an ApoE4-dependent manner, we employed both ApoE3-TR and ApoE4-TR female mice (4 months old), which were treated with FSH consecutively for 3 months. FSH treatment elevated C/EBPβ and its downstream AEP, which was proteolytically activated in ApoE4-TR mice. Subsequently, APP N585 and Tau N368 fragments and p-Tau were conspicuously augmented in ApoE4-TR as compared to ApoE3-TR mice (Fig. 4A, B). Accordingly, AEP enzymatic activities were much higher in ApoE4-TR mice than ApoE3-TR mice after FSH treatment (Fig. 4C). IF co-staining showed that FSH enhanced both p-Tau AT8 and Tau N368 signals in the cortex of ApoE4-TR mice as compared with ApoE3-TR mice (Fig. 4D). Furthermore, IF co-staining demonstrated that FSH strongly augmented both p-Tau and aggregated Tau T22 activities in the cortex and the hippocampus of ApoE4-TR mice versus ApoE3-TR mice (Fig. 4E, F). Fitting with these observations, Silver staining showed that FSH highly increased intra-neuronal proteinaceous inclusions in the CA1 region of the hippocampus, the cortex, the Entorhinal cortex (EC) (Fig. 4G). Thus, FSH selectively exacerbates AD pathologies in ApoE4-TR mice as compared to ApoE3-TR mice.

Golgi staining indicated that the dendritic spines were greatly reduced in ApoE4-TR as compared to ApoE3-TR mice upon FSH treatment (Supplementary Fig. 5B). Accordingly, EM analysis disclosed that FSH significantly attenuated the synapses in ApoE4-TR versus ApoE3-TR mice (Fig. 4H). Consistent with these findings, the synaptic proteins including PSD95, Synapsin and Synaptophysin were clearly reduced in ApoE4-TR mice versus ApoE3-TR mice (Supplementary Fig. 5C, D). In alignment with the extensive synaptic loss, MWM tests showed that FSH-elicited cognitive impairment trend in ApoE4-TR mice as compared to ApoE3-TR mice (Fig. 4I). Moreover, fear-conditioning tests also demonstrated that FSH elicited significant cognitive defects in ApoE4-TR mice compared to ApoE3-TR mice (Fig. 4J). Therefore, these findings strongly suggest that FSH induces greater cognitive dysfunction in ApoE4-TR mice compared to ApoE3-TR mice.

### Ovariectomy-triggered AD pathologies and cognitive disorders in ApoE4-TR mice via FSH

**Fig. 5:**
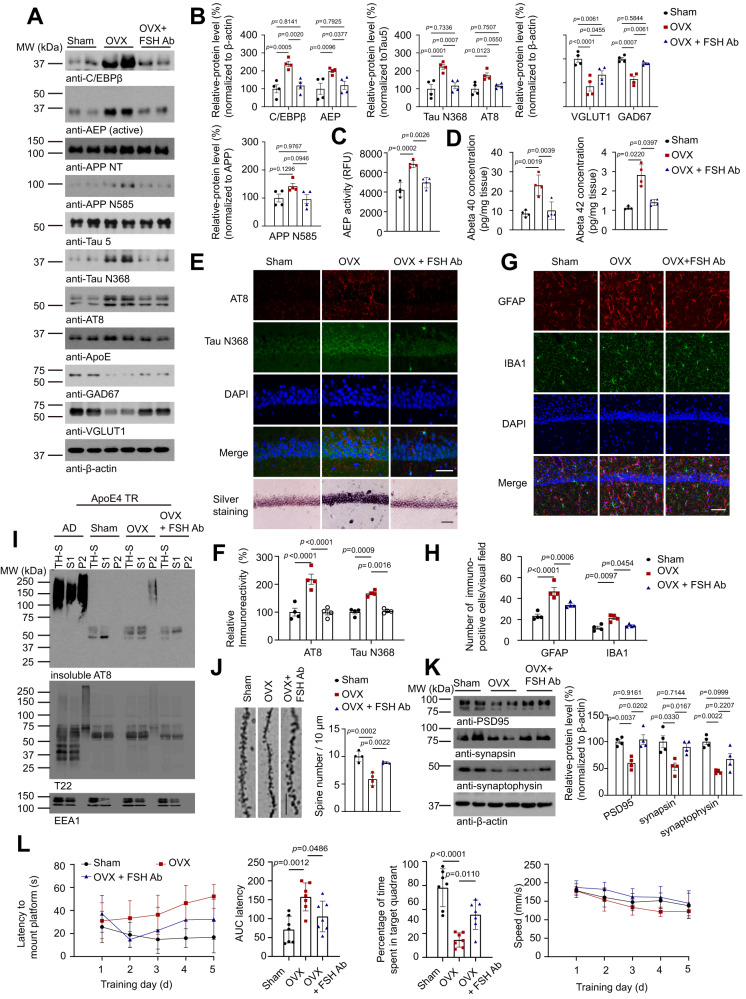
Ovariectomy triggers C/EBPβ/δ-secretase and AD pathology in female ApoE4-TR mice via FSH. Four months old female ApoE4 TR mice were subjected to sham or ovariectomy (OVX) operation, some of the mice after OVX were consecutively treated with anti-FSH antibody (FSH-Ab) (200 μg per day, i.p.) 4 days after OVX for 8 weeks (OVX + FSH Ab group). **A**, **K** Representative images of western blot showed the expression of C/EBPβ, AEP, cleaved APP, Tau and p-Tau, GAD67, VGLUT1, PSD95, synapsin, and synaptophysin in the hippocampus. **B**, **K** Quantification of protein expression. Data represent mean ± SEM (*n* = 4 mice per group, Two-way ANOVA for all the protein quantification except APP N585, one-way ANOVA followed by Tukey’s multiple comparisons test for APP N585). **C**–**H** AEP enzymatic activity (**C**), Aβ40 and Aβ42 concentration (**D**), AT8 (red)/Tau N368 (green) immune-reactivity (**E**, **F**), proteinaceous deposits (**E**) and GFAP (red) or IBA1 (green) positive cells (**G**, **H**) in the hippocampus were assessed. (scale bar = 50 μm (**E**), or 100 μm (**G**)). Data are shown as mean $\pm$ SEM. (*n* = 4 mice per group, one-way ANOVA followed by Tukey’s multiple comparisons test). **I** In order to show the aggregated Tau pathology, total homogenates (TH-S), Sarkosyl-soluble (S1) and Sarkosyl-insoluble (P2) fractions were blotted with antibody against AT8, or T22 for Tau oligomers, respectively. AD human cortex was a positive control. **J** Golgi staining showed the dendritic spine density. (scale bar = 10 μm). Data represent mean $\pm$ SEM (*n* = 4 mice per group, one-way ANOVA followed by Tukey’s multiple comparisons test). **L** Morris water maze analysis of cognitive functions of the ApoE4-TR mice after sham or OVX operation with or without FSH-Ab treatment. Data are shown as mean $\pm$ SEM (*n* = 7 mice per group, two-way ANOVA for latency, and speed analyze, one-way ANOVA for AUC latency, Time in target quadrant).

Ovariectomy (OVX) surgery disrupts female sex hormones and highly elevates FSH levels. FSH specific antibody prevents FSH binding to its receptor, antagonizing FSH/FSHR signalings^11^. Our recent studies showed that FSH antibody but not control IgG blocked p-C/EBPβ/AEP signaling in neurons, and anti-FSH antibody diminished OVX-provoked AD pathologies in 3xTg mice^39^. To explore whether OVX-stimulated FSH is implicated in ApoE4-mediated AD pathogenesis, we conducted OVX surgery in female ApoE3-TR and ApoE4-TR mice (4 months old), and half of the mice were treated with anti-FSH antibody or control IgG (200 μg per day, i.p.) 4 days after OVX consecutively for 8 weeks. Immunoblotting revealed that C/EBPβ expression was increased in female ApoE4-TR mouse brains after OVX as compared to sham control, so was the downstream target AEP. As a result, there’s an increase in APP N585, p-Tau and Tau N368, which was associated with increased FSH levels. As expected, blocking FSH with its specific antibody alleviated these events. Notably, OVX highly increased FSH in the brains as compared to sham. Interestingly, both GAD67 and VGLUT1 levels were clearly reduced in OVX-treated mice, which were alleviated by anti-FSH (Fig. 5A, B). AEP enzymatic activities were also increased by OVX, which was significantly reduced by FSH neutralization (Fig. 5C). Quantification showed that both Aβ40 and Aβ42 concentrations were substantially elevated in OVX-treated mice, which were strongly suppressed by FSH antibody (Fig. 5D).

IF co-staining showed that OVX strongly induced Tau N368 signals, accompanied with robust p-Tau AT8 activities in the hippocampus, which were diminished by anti-FSH antibody. In alignment with these findings, Silver staining also validated that OVX-triggered intensive proteinaceous inclusions in the hippocampus (Fig. 5E, F). T22 tightly correlated with AT8 activities, and we made the similar observations in the cortex, Dentate Gyrus (DG) regions (Supplementary Fig. 6A–D). Consistently, GFAP and Iba-1 co-staining demonstrated extensive astrogliosis and microglia activation triggered by OVX, and anti-FSH antibody alleviated these effects (Fig. 5G, H). To further characterize Tau aggregation, we conducted the ultra-centrifugation to separate total brain homogenate supernatant (TH-S) into different fractions. We employed human AD brains as a positive control, and found insoluble AT8-positive p-Tau proteins were detectable in both TH-S and S1 (soluble 1) fraction, but they were highly enriched in the P2 (pellet 2) fraction. By contrast, there was no any demonstrable insoluble p-Tau in the sham-ApoE4-TR mice, whereas they were detected in the P2 fraction from OVX-treated mice, which was eradicated after anti-FSH antibody treatment (Fig. 5I). Thus, these findings indicated that anti-FSH antibody effectively attenuated OVX-induced Tau pathologies in ApoE4-TR mice.

Golgi staining revealed the dendritic spine reduction in the hippocampal neurons upon OVX, which were alleviated by anti-FSH antibody (Fig. 5K). EM analysis showed that the synapses were greatly decreased in ApoE4-TR mice by OVX, which was rescued by anti-FSH antibody treatment (Supplementary Fig. 6E). Fitting with these findings, immunoblotting showed that synaptic proteins including PSD95, synapsin and synaptophysin were decreased by OVX, which were restored after anti-FSH antibody treatment. The MWM cognitive behavioral tests showed that OVX caused severe impairments in learning and memory, which was rescued by anti-FSH antibody, though these mice exhibited comparable swim speed, suggesting that the OVX surgery or anti-FSH antibody treatment does not affect their motor functions (Fig. 5L, Supplementary Fig. 6F). By contrast, there’s no significant AD pathology and cognitive dysfunction in ApoE3-TR mice (supplementary Fig. 7A–J). As a result of these data, OVX mimics menopause with high FSH levels, and induces the memory loss in ApoE4-TR mouse model, and blocking FSH with its antibody alleviates the effects by OVX. Hence, FSH mediates OVX-stimulated AD pathogenesis in female ApoE4-TR mice but not ApoE3-TR mice.

### OVX-induced FSH but not estradiol deficiency is responsible for stimulating AD pathologies

**Fig. 6:**
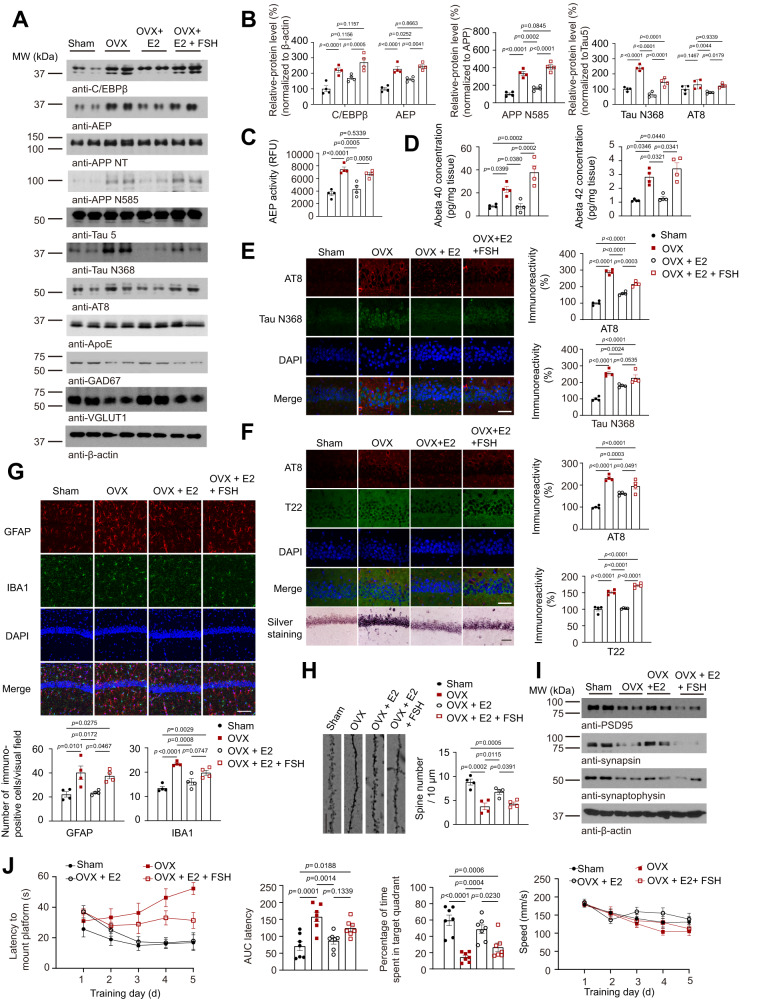
Ovariectomy-induced FSH but not estrogen triggers AD pathologies and cognitive deficits in female ApoE4-TR mice. At 4 months of age, female ApoE4-TR mice received sham or ovariectomy. After ovariectomy, some of the mice were subcutaneously embedded with a 90–day–release pellets (E2, 0.36 mg) of 17β-estradiol to render them biochemically eugonadal, and then with or without FSH treatment. The mice were randomly divided into four groups: sham, OVX, OVX + E2 and OVX + E2 + FSH. **A**, **I** Representative images of western blot showed the expression of C/EBPβ, AEP, cleaved APP, Tau, p-Tau, cleaved Tau, GAD67, VGLUT1, PSD95, synapsin and synaptophysin in the hippocampus. **B** Quantification of the protein expression. Data are shown as mean $\pm$ SEM. (*n* = 4 mice per group, two-way ANOVA or one-way ANOVA followed by Tukey’s multiple comparisons test). **C**, **D** AEP enzymatic activity (**C**), Aβ40, and Aβ42 concentration (**D**) in the hippocampus were examined. Data are presented as mean ± SEM. (*n* = 4 mice per group, one-way ANOVA followed by Tukey’s multiple comparisons test). **E**–**G** Immunofluorescent (IF) staining showed AT8 (red)/Tau N368 (green) (**E**), AT8 (red)/T22 (green) immuno-reactivity (**F**), and GFAP (red) or IBA1 (green) positive cells (**G**) in the hippocampus. Silver Staining showed the proteinaceous deposits in the CA1 regions (**F**). (scale bar = 50 μm (**E**, **F**), 100 μm (**G**)). Data are shown as mean ± SEM (*n* = 4 mice per group, one-way ANOVA followed by Tukey’s multiple comparisons test)). **H** Golgi staining showed the dendritic spine density in CA1. (scale bar, 10 μm). Data represent mean $\pm$ SEM (*n* = 4 mice per group, one-way ANOVA followed by Tukey’s multiple comparisons test). **J** Morris water maze analysis of cognitive functions showed E2 supplement alleviated OVX-induced learning and memory impairments, but FSH injection after E2 supplement augmented learning and memory impairments. Data shown as mean $\pm$ SEM (*n* = 7 mice per group, two-way ANOVA for latency, and speed analyze, one-way ANOVA for AUC latency, time in target quadrant).

Estrogen therapy in postmenopausal women has been suggested to be beneficial for both early and late onset AD^40,41^, though extensive inconsistent data regarding the effect of ERT (estrogen replacement therapy) on AD have been reported. During peri-menopause, circulating FSH levels increase, along with a drop in estrogen^42,43^. It is crucial to maintain normal estrogen levels to rule out the effects of estrogen on C/EBPβ/AEP signaling because there is a feedback regulation loop between FSH and estrogen and a drop in estrogen level may influence AD pathology. Therefore, we performed OVX surgery on ApoE4-TR female mice and kept their E2 levels normal by supplementing them with exogenous estrogen (OVX + E2). And then, exogenous FSH was additionally administered into the mice (OVX + E2 + FSH) to raise FSH levels up to the concentration comparable to OVX group. The OVX + E2 + FSH mice that received FSH showed normal E2 levels in addition to elevated FSH levels, mimicking the traits of peri-menopausal women. As expected, OVX surgery induced evident uterus atrophy in female ApoE4-TR mice as compared to sham control, which was alleviated in the presence of E2 (Supplementary Fig. 8A). Consequently, FSH concentrations were greatly enhanced, while estradiol levels were dramatically decreased in the serum of OVX-treated mice (Supplementary Fig. 8B). The OVX + E2 mice showed diminished C/EBPβ/AEP signaling as compared to the OVX group, which resulted in lessened APP N585 and Tau N368 fragmentation by active AEP, which was correlated with decreased p-Tau AT8 signals. As expected, VGLUT1 and GAD67 levels were also rescued by exogenous E2 as compared to OVX group. These findings suggested that E2 might repress OVX-induced FSH, inhibiting its stimulatory effect on C/EBPβ/δ-secretase pathway. Consequently, supplementing FSH in (OVX + E2 + FSH) group reversed these events (Fig. 6A, B). AEP enzymatic activity assay mirrored active AEP levels in IB (Fig. 6C). Subsequently, ELISA quantification revealed that both Aβ40 and Aβ42 echoed APP N585 fragmentation activities (Fig. 6D). Thus, these findings support that OVX-induced FSH but not E2 deficiency accounts for the C/EBPβ/AEP signaling activation and the downstream AD pathological effects. IF showed that Tau N368/AT8 co-staining activities matched IB observation, oscillating with FSH concentrations (Fig. 6E). Consistently, AT8/T22 co-staining demonstrated higher activities of T22 in both OVX group and (OVX + E2 + FSH) group, coupled with robust AT8 signals, as compared to sham or OVX + E2 group (Fig. 6F). Silver staining also confirmed OVX-provoked proteinaceous inclusions in the hippocampus, which were attenuated by E2 supplement. Introduction of exogenous FSH in (OVX + E2 + FSH) groups partially restored the protein aggregation (Fig. 6F, bottom panel). In the cortex and DG areas, we made comparable observations. (Supplementary Fig. 8C, D). Moreover, GFAP/Iba-1 co-staining also validated OVX-induced FSH mediated astrogliosis and microglia activation (Fig. 6G).

Golgi staining showed that OVX-treated female ApoE4-TR mice exhibited significantly reduced synaptic dendrites as compared to sham group, which were increased in (OVX + E2) mice. Again, FSH supplement substantially resulted in dendritic spines reduction in (OVX + E2 + FSH) mice (Fig. 6H). Furthermore, EM also demonstrated that OVX-induced FSH but not E2 scarcity accounted for the synapse degeneration in female ApoE4-TR mice incurred by OVX (Supplementary Fig. 8E). In agreement with these observations, IB of the synaptic proteins including PSD95, Synapsin and Synaptophysin demonstrated the similar fluctuating pattern (Fig. 6I). As a result, OVX-triggered FSH-elicited cognitive deficits in ApoE4-TR mice were clearly alleviated in (OVX + E2) mice. Again, introduction of exogenous FSH restored the memory dysfunctions in (OVX + E2 + FSH) mice (Fig. 6J). Together, these studies strongly support that FSH or ovariectomy, independent of estrogen, triggers AD pathologies and cognitive defects via activating C/EBPβ/δ-secretase pathway.

### Neuronal but not astrocyte-derived ApoE4 is essential for AD pathology in the presence of FSH

**Fig. 7:**
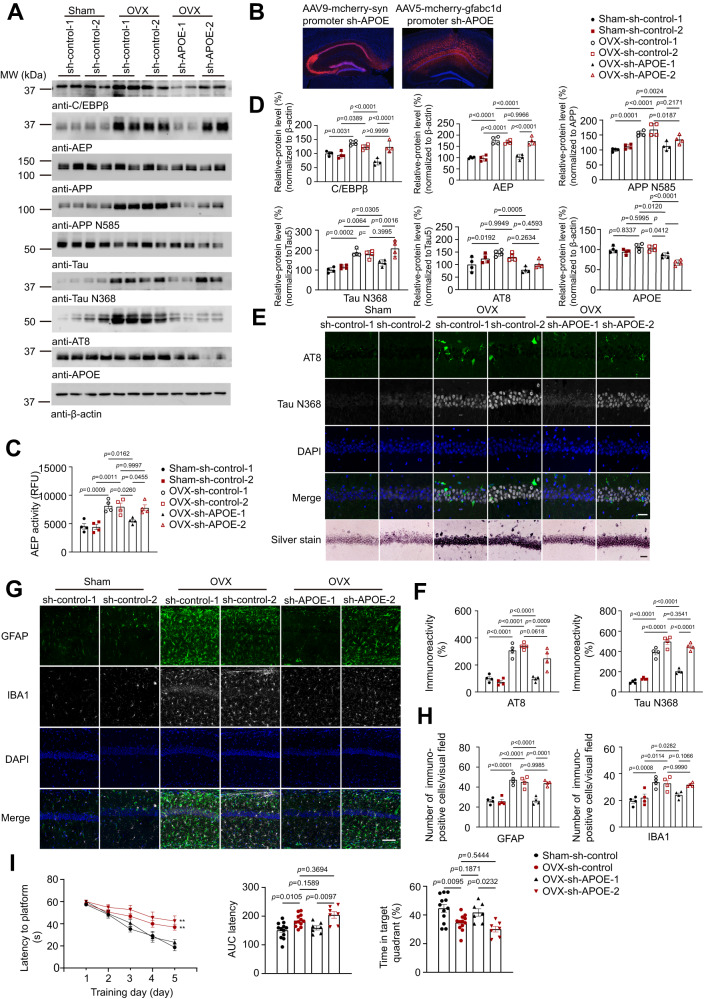
Neuronal but not glia ApoE4 and FSH jointly promote AD pathology. AAV9-syn-sh-APOE virus or AAV5-gfabc1d-sh-APOE virus was utilized to separately knock-down APOE expression in the hippocampus neurons or astrocytes in female ApoE4-TR mice before OVX. AAV9-syn-sh-control virus, AAV5-gfabc1d-sh-control virus, AAV9-syn-sh-APOE virus and AAV5-gfabc1d-sh-APOE virus are abbreviated as sh-control-1, sh-control-2, sh-ApoE-1 and sh-ApoE-2 respectively. **A** Representative images of western blot showed that depletion of neuronal ApoE but not glial ApoE prominently diminished C/EBPβ/AEP signaling triggered by OVX, leading to suppression of APP N585 and Tau N368 fragmentation. **B** Virus expression (red) in the hippocampus. **C** Brain AEP enzymatic activity was examined. Data are shown as mean $\pm$ SEM. (*n* = 4 mice per group, one-way ANOVA followed by Tukey’s multiple comparisons test). **D** Quantification of western blot data (*n* = 4 mice per group, one-way ANOVA followed by Tukey’s multiple comparisons test). **E** IF staining showed AT8 (green) and Tau N368 (grey) in the hippocampus. Silver Staining showed the proteinaceous deposits in CA1 regions. (scale bar, 50 μm). **F** Quantitative of AT8 and Tau N368 immunoreactivity. Data are presented as mean ± SEM. (*n* = 4 mice per group, one-way ANOVA followed by Tukey’s multiple comparisons test). **G** IF staining showed GFAP (green) and IBA1 (gray) positive cells in the hippocampus (scale bar, 100 μm). **H** Quantification of GFAP and IBA1 positive cells. Data are presented as mean ± SEM (*n* = 4 mice per group, one-way ANOVA followed by Tukey’s multiple comparisons test). **I** Morris water maze analysis of cognitive functions. Data are shown as mean $\pm$ SEM (*n* = 7 or 12 mice per group, two-way ANOVA for Latency, one-way ANOVA for AUC latency, Time in target quadrant, ** *p* ≤ 0.01).

ApoE can be produced in glial cells, neurons, and other types of cells in the CNS. It is not clear whether ApoE4 made in glial cells or neurons (or both) is important for additively affecting AD pathologies with FSH. To address this question, we employed AAV9-syn-sh-APOE virus or AAV5-gfabc1d-sh-APOE virus to separately knock-down the APOE expression in neurons or astrocytes the hippocampus before OVX. IF staining revealed that both neuronal and glial ApoE levels were pronouncedly decreased by the virus in the hippocampus. We found that depletion of neuronal ApoE but not glial ApoE prominently diminished C/EBPβ/AEP signaling triggered by OVX, leading to suppression of APP N585 and Tau N368 fragmentation (Fig. 7A, B). These were associated with reduced AT8 signals and decreased AEP enzymatic activities (Fig. 7C, D). These effects were also validated by IF co-staining with AT8 and Tau N368 antibodies. Silver staining confirmed that OVX-elicited proteinaceous inclusions were more prominently alleviated by neuronal ApoE depletion than glial ApoE deletion (Fig. 7E, F). IF co-staining demonstrated that OVX-stimulated AT8 and T22 activities were substantially abolished when neuronal ApoE was deleted compared to glial ApoE depletion (Supplementary Fig 9A). In alignment with these observations, EM analysis revealed that OVX-surgery elicited evident synaptic loss, which was alleviated by neuronal but not glial ApoE depletion (Supplementary Fig. 9B). As a result, both GFAP and Iba1 staining signals triggered by OVX were robustly repressed when neuronal ApoE was deleted; in contrast, these effects were barely attenuated when glial ApoE was eradicated (Fig. 7G, H). Golgi staining showed that OVX-elicited significant dendritic spines loss upon OVX versus sham control group, which was significantly ameliorated by neuronal but not glial ApoE knockdown in the hippocampus (Supplementary Fig. 9C). MWM cognitive assay showed that inactivation of neuronal ApoE restored cognitive functions as compared to glial ApoE knockdown group (Fig. 7I, Supplementary Fig. 9D). Hence, these findings demonstrate that ApoE in neurons is much more important for additively exacerbating AD pathologies by FSH than glia counterpart.

## Discussion

Although the impact of sex on AD epidemiology is currently the focus of significant research, the concept of sex-specific pathogenic AD phenotypes remains little explored. In this study, we provide compelling evidence supporting that FSH and ApoE4 additively activate C/EBPβ/δ-secretase signaling both in AD mouse brain and primary culture neurons (Fig. 1). FSH activates p-C/EBPβ T188 in cells, which is mediated by p-MAPK^20^. Most recently, we reported that FSH stimulates δ-secretase activation via Akt-mediated SRPK2 phosphorylation of δ-secretase S226^39^, which escalates δ-secretase protease activities and translocates it from the lysosomes into the cytoplasm^44^. FSH is long thought to exert its effects in gonadal tissues, mainly limited to Sertoli cells in testes and granulosa cells in ovaries. And its cognate receptor FSHR is expressed in extragonadal tissues, including endothelium, monocytes, developing placenta, bone and fat^45^. We have shown that FSHR is expressed in neurons in the human and mice brain^39^. FSH activates C/EBPβ/δ-secretase pathway and δ-secretase protease activities in a dose-dependent manner in human iPSC-derived neurons, which subsequently cleaves both APP and Tau at N585 and N368 residues, respectively (Fig. 1 and Fig. S1). To assess whether FSH elevation accelerates AD pathology onset, we administrate FSH into both young male and female ApoE4-TR mice, and find that FSH activates C/EBPβ/δ-secretase signaling and prominently facilitates the NFT pathologies in the brain from both sexes as compared to vehicle control (Figs. 2 and 3). Remarkably, these effects selectively occur to ApoE4 but not ApoE3-TR mice (Fig. 4). Moreover, we employed ovariectomy to increase FSH levels in ApoE4-TR mice and disclose the same demonstrable AD pathologies, which are robustly abrogated by neutralizing FSH via its specific antibody (Fig. 5). Consequently, FSH administration strongly impairs cognitive functions in both male and female mice, whereas anti-FSH therapy potently ameliorates the ovariectomy-induced cognitive disorders (Fig. 5). It is worth noting that the learning curves for the OVX-treated mice were unusual in Figs. 5L and 6J. During the training course, the swimming distances by the OVX mice correlate with the latency to the mount platform. We included the distance data in Supplementary Figs. 6F and 8F, and we did see the learning improvement across the five days. Noticeably, the swimming speeds of the OVX mice are slightly different from other groups. Previous studies show that menopausal transition exposes women to an early decline in muscle force and motor function^46^. The muscle twitch force potentiation is negatively associated with FSH levels. Like menopaused women, OVX female mice displayed impaired motor functions as the swim speed is abnormally lower than sham or OVX-FSH Ab group (Figs. 5L and 6J). Conceivably, the aberrant learning behavior curves may be related to strange motor dysfunction, which is associated with elevated FSH levels. Furthermore, the OVX-treated mice received exogenous E2 supplementation to keep their E2 levels similar to Sham control mice in order to avoid the potential impact of estrogen deprivation. E2 feedback at the pituitary gland also caused endogenous FSH levels to be unstable under these conditions. With this approach, it was possible to manipulate the FSH levels by administering exogenous FSH without altering the E2 levels. Our data support that OVX-induced FSH but not estrogen paucity mediates C/EBPβ/δ-secretase signaling, attributing to AD pathogenesis in ApoE4-TR mice (Fig. 6). Hence, these compelling findings provide the provocative evidence supporting that FSH and ApoE4 jointly mediate AD pathogenesis in female via activating C/EBPβ/δ-secretase pathway.

The prevalence of AD is significantly higher in women compared to men, about two thirds of the patients diagnosed with AD are women. Female sex is one of the major risk factors for developing late-onset AD^47^. Nevertheless, the molecular mechanism accounting for this effect remains unclear, though intensive efforts have been made. Sex may play a significant role in stratifying AD patients and determining their treatment due to the emerging evidence of sex-related differences in brain structure and function. For instance, as compared to men, women with AD experience different cognitive and psychiatric symptoms, and they show faster cognitive decline following the diagnosis of mild cognitive impairment (MCI) or AD dementia, despite the fact that there are no sex differences in the levels of amyloid-based brain imaging or the biochemical analysis of cerebrospinal fluid. Furthermore, there are sex-specific differences in the rates and patterns of brain atrophy along the AD continuum; in MCI, women experience brain atrophy more quickly than men^48^. A series of study have found that bilateral or unilateral oophorectomy is associated with the increased risk of cognitive decline or dementia^15–17^. An abrupt decline in circulating estrogen level has been reported after bilateral oophorectomy, resulting in degenerative and vascular brain lesions^49^. Cognitive impairment or dementia may develop several decades after an oophorectomy caused by these lesions in the brain. Clinical trials show that women aged 65-79 who receive estrogen alone or estrogen plus a progestin have an increased risk of cognitive impairment and dementia^50^, which is a indirect evidence that the feedback inhibition of FSH by estrogen supplementation in early perimenopause may be therapeutic for cognitive decline.

In the ovary, FSH regulates folliculogenesis, oocyte selection, and the synthesis of sex steroid hormones, thus preparing the reproductive tract for fertilization, implantation, and pregnancy^51^. FSH is considered as a primary stimulus for estrogen production from ovarian follicular cells^52^. As FSH secretion is negatively regulated by estrogenic feedback, a high circulating FSH level invariably accompanies hypogonadism. FSH has thus been regarded as a marker for the onset of menopause^53^. To alleviate the concern regarding strong implicated in ovariectomy-elicited AD pathologies, we also include estrogen or estrogen + FSH in the ovariectomy-treated female ApoE4-TR mice and find that it fails to attenuate AD pathologies onset and progression in these mice (Fig. 6), suggesting that FSH but not estrogen is accountable for triggering AD pathologies. These findings are in alignment with previous reports that it is the pituitary-derived hormone FSH, rather than serum estrogen, that correlates best with markers of bone turnover in postmenopausal women^54^. Due to the predisposition of women to AD, the sex steroid estrogen has become the primary focus of research in this field, however, inconclusive data regarding ERT triggers further scrutiny of the role of the other hormones of the hypothalamic-pituitary-gonadal (HPG) axis. For instance, it has been postulated that the estrogen produced by the ovaries in a woman before the onset of menopause has an important neuroprotective effect on the brain^49^. It has been proposed that the hormones of the HPG axis, such as the gonadotropin, (luteinizing hormone (LH) and FSH), are involved in regulating reproductive function via a complex feedback loop. The increase in gonadotropin concentrations and not the decrease in steroid hormone (e.g., estrogen) production following menopause that might result in an increased risk of AD^55^.

Apolipoprotein E series, especially APOE ε4 allele, is the major genetic risk factor for sporadic and familial AD, increases the risk for both early-onset and late onset AD. Women APOE ε3/ε4 genotype display an increased risk of AD or developing MCI compared with men^1^. In APOE4-KI mice, female not male mice exhibit age-dependent decrease in the numbers of hilar GABAergic interneurons^56^. In alignment with these observations, we show that GAD67 levels are robustly reduced in OVX-treated mice and FSH is predominantly accountable for this effect (Figs. 5 and 6, Supplementary Fig. 4). In our most recent study, we demonstrated that C/EBPβ plays a crucial role as a transcription factor for ApoE, and ApoE4 feeds back and activates C/EBPβ/δ-secretase pathway in the presence of 27-hydroxycholesterol, driving AD pathogenesis^57,58^. Employing AAV9 and AAV5 virus expressing shRNA to selectively depleting ApoE in neurons or astrocytes, we find that neuronal but not glial ApoE4 is essential for jointly activating this pathway with FSH (Fig. 7, Supplementary Fig. 9). Our study further confirms the toxic role of neuronal ApoE in AD pathogenesis^59,60^. Consequently, neuronal ApoE4 stimulates C/EBPβ activation in Thy1-ApoE4/C/EBPβ transgenic mice, promoting AD pathologies via mouse machinery^61^. Thus, both ApoE4 and FSH stimulate C/EBPβ/δ-secretase pathway activation in the brain. Together, our results indicate APOE4 and female sex are synergistic risk factors in AD pathogenesis. Conceivably, dissecting the molecular relationship between FSH and ApoE4 and their roles in activating C/EBPβ/δ-secretase signaling pathway, selectively triggering AD pathogenesis in female, will provide significant insight into the molecular mechanism and potential drug targets for interfering the devastating neurodegenerative disease.

## Methods

### Animals and treatment

#### Mice

ApoE4-Targeted Replacement (ApoE4-TR) and ApoE3-Targeted Replacement (ApoE3-TR) mice were obtained from TACONIC (stock# 1549 and 1548, respectively). All mice were housed at a temperature of 22 °C with a 12-h/12-h light/dark cycle while being kept in a pathogen-free environment. Water and food were available as needed. The tests were carried out in accordance with the Emory School of Medicine’s guidelines and the NIH animal care guidelines for animals. The Institutional Animal Care and Use Committee (IACUC) at Emory University evaluated and approved the procedure. Four months old mice were used in this experiment.

#### FSH treatment

The 4-month-old female, male ApoE4-TR and female ApoE3-TR mice were received PBS or recombinant human FSH (5 IU, per day, 6 days a week) via intraperitoneal injection for 3 months.

#### Ovariectomy, and with E2, FSH or anti-FSH antibody supplement

The four months old female ApoE4 TR mice were anesthetized with 2% isoflurane and placed on a warm pad. According to the methodology approved by the Emory Institutional Animal Care and Use Committee, female ApoE4 TR mice were randomly assigned with either sham operation (sham) or bilateral ovariectomy (OVX) after a midline dorsal incision (~2 cm). After the OVX, some of the mice were implanted with 17β-estradiol (E2, 0.36 mg, 90 days release). These mice were randomly divided into 5 groups: sham, OVX, OVX + E2, OVX + E2 + FSH and OVX + FSH-Ab group. These mice were allowed 4 days recover after OVX, the OVX + E2 and OVX + E2 + FSH group mice were received PBS or recombinant human FSH (5IU/d, 6 days a week) via intraperitoneal injection for 8 weeks. In OVX + FSH-Ab group, the mice were received intraperitoneal injections of anti-FSH antibody (200 μg/d, 6 days a week) for 8 weeks.

#### Stereotactic injection

Female ApoE4-TR mice (aged 4 months) were stereotactically injected with AAV9-syn-sh-APOE virus, AAV5-gfabc1d-sh-APOE virus, and the control virus from the BrainVTA Co., Ltd. For bilateral intracerebral injections, we selected the following coordinates: dorsoventral distance of −1.5 mm, −1.8 mm mediolateral and −2.1 mm anteroposterior from the bregma. A 0.2 μl viral solution containing 2 × 10^12^ vector genomes per ml was injected into each site at a speed of 0.1 μl per minute using a 10 μl glass syringe with an immovable needle. A needle was kept for five minutes, and then gradually removed over two minutes. A heating pad was used to keep the body temperature of the mice until they changed positions from supine to prone, which is a sign that they were recovering from anesthesia. Seven days following the stereotactic injection, the mice underwent ovariectomy.

### Lipidated ApoE production

Lipidated ApoE3, and ApoE4 proteins were produced both in HEK293T cells according to the method of Huang et al.^62^ with minor modification. HEK293T cells were cultured at 37 °C, 5% CO_2_ in DMEM supplemented with 10% (vol/vol) FBS. Cells were plated at 250,000 cells/mL and transfected 24 h later with Lipofectamine 3000 and 5 μg of DNA for APOE3 or APOE4 tagged with EGFP. The medium was changed 24 h after transfection, followed by washing once with PBS, and then incubated for 48 h in serum-free DMEM. Supernatants from transfected 293 T cells were harvested to determine the purity and yields of recombinant ApoE proteins. To assess the lipidation state of APOE in the medium, 20 μL of conditioned medium from APOE transfected HEK293T cells was added to 6.25 μL Native Gel Sample Loading Buffer (Beyotime P0016N) and run on a NativePAGE gel (Beyotime P0545S) in NativePAGE Running Buffer (Beyotime P0556). To assess APOE concentration, 20 μL of medium was analyzed by SDS/PAGE. After SDS/PAGE or Native-PAGE, the samples were transferred to a nitrocellulose membrane. The membrane was blocked with TBS containing 5% nonfat milk and 0.1% Tween 20 (TBST) at room temperature for 2 hours, followed by the incubation with primary antibody at 4 °C overnight, and with the secondary antibody at room temperature for 2 hours. After washing with TBST, the membrane was developed using the enhanced chemiluminescent (ECL) detection system. The ApoE supernatants were pooled and concentrated to 2 mL using a 10 kDa MWCO Amicon® Ultra-15 mL Centrifugal Filter Unit (Merck). The concentrated sample were diluted to proper concentration by PBS and added into cultured neurons for ApoE stimulation.

### Primary rat neuron culture

Primary culture rat cortical neurons were described in previous paper^63^. To determine the effect of FSH, ApoE3 and ApoE4 on neurons, primary cultured cortical neurons (DIV 13) were treated with vehicle, FSH (30 ng/ml), recombinant human ApoE3 (100 mM), ApoE4 protein (100 mM), lipidated ApoE3 or ApoE4 proteins for 48 hours. Then the neurons were harvested for western blot, AEP activity assay, or fixed in 4% formaldehyde, permeabilized and immunostained with anti-C/EBPβ, anti-AEP, anti-Aβ, anti-APP C586, anti-AT8, anti-Tau N368 and anti-ApoE. The supernatants were collected for LDH assay (Promega J2380).

### Differentiation of human iPSC-derived NSCs into neurons

NSCs derived from human induced pluripotent stem cells (iPSCs) were cultured and differentiated into mature neurons as described previously^57^. The iPSC-derived NSCs were obtained from two donors: one with ApoE4/4 genotype (ax0111) and the other one with ApoE3/3 genotype (ax0112) (Axol Bioscience, Cambridge, UK). And we use another line of human iPSC to do the similar experiment, which was from Accegen Biotechnology, USA. The PLO/Laminin-coated plates were used as the culture plate in the differentiation of NSCs into neurons. And then the cells were cultured in neuronal differentiation medium, which consists of DMEM/F12 + Neurobasal Medium (1:1), GDNF (20 ng/ml), B27, BDNF, IGF (10 ng/ml), NT3 (10 ng/ml), N2(20 ng/ml), ascorbic acid (200 μM) (all from Stemcell Technologies), and dbcAMP (100 nM) (Sigma-Aldrich). During in vitro maturation, the neuronal culture medium was changed once after 48 h to remove unattached cells. After that, a half change was made every 3 days. After 6 weeks of differentiation, the cells were fixed in 4% formaldehyde, permeabilized and co-stained with anti-MAP2 and Tuj1 for validation. The cells were then exposed to various FSH concentrations for 48 hours.

### Regents and materials

Antibody to C/EBPβ (HT-7) (catalog#: sc-7962, 1:200 for immunofluorescence and 1:1000 dilution for Western blot) was from Santa Cruz; anti-AEP (6E3) was a gift from Dr. Colin Watts (1:1000 dilution for Western blot), Professor of Immunobiology, Division of Cell Signaling and Immunology, College of Life Sciences, University of Dundee, Dundee, UK; antibodies to phosphor-Tau (Ser202-Thr205) (AT8, catalog#: MN1020, 1:300 for immunofluorescence and 1:1000 dilution for Western blot), phosphor-Tau (Thr212, Ser214) (AT100, catalog#: MN1020, 1:200 for immunohistochemistry) and IBA1 (catalog#: PA5-18039, 1:400 dilution for immunofluorescence) were from Thermo Fisher Scientific; antibodies to AEP (D6S4H) (catalog#: 93627, 1:500 for immunofluorescence and 1:2000 dilution for Western blot), PSD95 (catalog#: 2507, 1:1000 dilution for Western blot) and synapsin (catalog#: 5297, 1:1000 dilution for Western blot)were obtained from Cell Signaling Technology; antibody to Aβ (4G8) (catalog#: 800701, 1:200 dilution for immunofluorescence) was obtained from Biolegend; antibody to Tau 5 (catalog#: MAB361, 1:2000 dilution for Western blot), β-actin (catalog#: A5316, 1:3000 dilution for Western blot) and GFAP (catalog#: MAB360, 1:400 dilution for immunofluorescence)were from Sigma-Aldrich; antibody to ApoE (catalog#: AB947, 1:1000 dilution for Western blot), T22 (catalog#: ABN454, 1:700 dilution for immunofluorescence) and GAD67 (catalog#: MAB5406, 1:2000 dilution for Western blot and 1:500 dilution for immunofluorescence) was bought from Millipore Sigma; antibody to VGLUT1 (catalog#: 135311, 1:500 dilution for immunofluorescence) were from Synaptic Systems; antibody to synaptophysin (catalog#: ab32127, 1:1000 dilution for Western blot) were from Abcam; antibodies to Tau N368 (1:3000 dilution for Western blot and 1:700 for immunofluorescence) and APP N585 (1:1000 dilution for western blot) were produced in the Ye lab. Human ApoE3 (catalog#: 350-02) and ApoE4 (catalog#: 350-04) recombinant protein were purchased from ThermoFisher Scientific. The anti-FSHβ polyclonal antibody (FSH Ab) was developed and characterized in the Zaidi lab. Human Aβ40 (catalog#: KHB3481), Aβ42 (catalog#: KHB3544), mouse Aβ40 (catalog#: KMB3481) and mouse Aβ42 (catalog#: KMB 3441) were purchased from Invitrogen. sta. Z-Ala-Ala-Asn-AMC is the AEP substrate from Bachem (catalog number 4033201). Recombinant human FSH was purchased from EastCoast Bio (Catalog#: LA252) and Sigma-Aldrich (Catalog#: F4021) for the in vivo and in vitro experiments respectively. The following 90–day–release pellets containing 0.36 mg 17β-estradiol (catalog#: NE121) were obtained from Innovative Research of America. The AAV9-syn-sh-APOE virus, AAV5-gfabc1d-sh-APOE virus, AAV9-syn-sh-control virus and AAV5-gfabc1d-sh-control virus (2 × 10^12^ vector genomes per ml) were obtained from BrainVTA Co., Ltd. (Wuhan, China). Sigma-Aldrich supplied all chemicals not mentioned above.

### AEP activity assay

Tissue homogenates or cell lysates (10 μg) were incubated in 200 μl assay buffer (20 mM citric acid, 60 mM Na_2_HPO_4_, 1 mM EDTA, 0.1% CHAPS, and 1 mM DTT, pH 6.0) containing 20 μM δ-secretase substrate Z-Ala-Ala-Asn-AMC (Bachem). In a fluorescence plate reader, we measured the AMC released by substrate cleavage at 460 nm for two hours in kinetic mode at 37 °C

### Enrichment of detergent-insoluble protein aggregates

Enrichment of detergent- insoluble protein aggregates from tissue was performed as previous^64^. Briefly, mouse brain tissues were homogenized in low salt buffer with protease and phosphatase inhibitor cocktail. The homogenate was transferred to a tube and labeled with “TH”, and mixed with 5 M NaCl and 10% (w/v) sarkosyl to concentrations of 0.5 M and 1% w/v, and then incubated on ice for 15 min. The solution was labeled with “TH-S” (Total Homogenate-Sarkosyl), which indicated that the homogenates were in the sarkosyl-buffer. The tube was sonicated and then TH-S homogenates were diluted to 10 mg/ml using ice-cold sark buffer, ultracentrifuged at 180,000 × *g* for 30 min at 4 °C. The supernatants were labeled as S1 (Sarkosyl soluble). After pulse-spun for 2–3 s (≤2500 × *g*), the insoluble pellets (P1) were resuspended in sark-buffer, and then centrifuged at 180,000 × *g* for 30 min at 4 °C. The supernatant was discarded and the Sarkosyl-insoluble pellets (P2) was incubated in the urea buffer for 30 min at room temperature to solubilize the pellet. The protein concentrations of Total Homogenate-Sarkosyl (TH-S), the sarkosyl-soluble (S1) and -insoluble (P2) fractions were determined and run on SDS-PAGE gels.

### Western blot analysis

Cells and brain samples tissue were washed with ice-cold PBS and lysed in lysis buffer (50 mM Tris, pH 7.4, 40 mM NaCl, 1 mM EDTA, 0.5% Triton X-100, 1.5 mM Na_3_VO_4_, 50 mM NaF, 10 mM sodium pyrophosphate, 10 mM sodium β-glycerophosphate, supplemented with protease inhibitors cocktail) at 4 °C for 0.5 hours, and centrifuged for 25 min at 15,000 rpm. The supernatant was boiled in the SDS loading buffer. The samples were transferred to a nitrocellulose membrane after SDS-PAGE. The membrane was blocked with TBS containing 5% nonfat milk and 0.1% Tween 20 (TBS-T) at room temperature for 2 hours, followed by the incubation with primary antibody at 4 °C overnight, and with the secondary antibody at room temperature for 2 hours. The enhanced chemiluminescent (ECL) detection system was used to develop the membrane after washing with TBST.

### Immunostaining

The free-floating 25 μm brain sections were used in immunostaining. For IHC staining, the brain sections were treated with 0.3% H_2_O_2_ for 10 min. Then sections were washed three times in PBS and blocked in 1% BSA and 0.3% Triton X-100, for 30 min followed by overnight incubation with anti-AT100 (1:400) at 4 °C. An IHC kit specific to Rabbit and Mouse HRP/DAB detection was used to detect the signal (Abcam). For immunofluorescence staining, the brain sections were incubated with primary Aβ, APP C586, AT8, Tau N368, T22, GFAP, IBA1, VGLUT1 and GAD67 antibodies overnight at 4 °C after blocking in blocking buffer for 1 hours at room temperature. After washing with Tris-buffered saline, the sections were incubated with a mixture of Alexa Fluor 488- and 594-coupled secondary antibodies (Invitrogen) for detection. DAPI (1 μg/mL) (Sigma) was used for staining nuclei. Images were acquired with an Olympus confocal FV1000 imaging system. ImageJ (NIH) was used for intensity analysis.

### Gallyas Silver staining

Silver staining was performed using the Gallyas method. The alkaline silver iodide solution (containing 1% silver nitrate) was applied to brain sections (25 m) for 1 minute after they had been treated in 5% periodic acid for 5 minutes. After being washed in 0.5% acetic acid for 10 minutes, the sections were then dipped in developer solution for 15 minutes before being once more washed in 0.5% acetic acid for 10 minutes and once more in water. The sections underwent a 5 minutes treatment with 0.1% gold chloride, followed by a water wash and a 5 minutes incubation with 1% sodium thiosulfate (hypo) before one final washing.

### Golgi stain

After being fixed in 10% formalin for 24 hours, mouse brains were submerged in 3% potassium bichromate for 3 days in the dark. Every day, a new solution was proposed. After been transferred into 2% silver nitrate solution, the brains were incubated in the dark for 7 days. Every day, a new solution was proposed. After cutting 30 mm sections with a vibratome, and then the sections were air dried for 10 minutes, dehydrated by 95% and 100% ethanol, cleared in xylene, and finally sealed with coverslips.

### Morris water maze

The morris water maze test was described as previous^31^. Mice were trained in a 52-inch-diameter, circular, water-filled tub in a setting full of additional maze signals. For five days training, each individual received four trials each day, with a 15-minute break between each trial. The maximum trial duration was 60 second, and the subjects were manually guided to the platform if they were unable reach the platform in the allotted time. Following five days of task acquisition, a probe trial was conducted. During this trial, the platform was removed, and over the course of 60 seconds, the percentage of time spent in the quadrant that had previously contained the escape platform during task acquisition was calculated. Using MazeScan (Clever Sys, Inc.), latency and swim speed were examined for each trial.

### Fear condition test

The fear condition test was described as previous paper^31^. Three-day fear condition paradigms were used to test the ability to form and retain associations between aversive experiences and environment cues. In a fear condition apparatus (7″ W, 7″ D × 12″ H, Coulbourn), mice explored the enclosure for 3 minutes with a metallic shock grid floor. Following this habituation period, 3 conditioned stimulus (CS)-unconditioned stimulus (US) pairings was presented with a 1 min intertrial interval. The CS was composed of a 20 second 85 db tone and US was composed of 2 s of a 0.5 mA footshock, which was co-terminate with each CS presentation. The mice were brought back to their original cage one minute after the final CS-US presentation. A context test was administered to the mice on day 2, with subjects being put in the identical chamber as before.

### Statistical analysis

GraphPad Prism 9.1.0 (GraphPad Software, Inc) and Image J 1.51J8 were used for analysis. GraphPad Prism 9.1.0 was used for statistical analysis and data was analyzed using either Student’s *t* test (two-group comparison), one-way or two-way ANOVA followed by a post hoc test (Tukey’s) (multigroup comparisons) which was indicated in the figure legend. There was considered to be a significant difference when the *P* value was <0.05.

### Reporting summary

Further information on research design is available in the Nature Portfolio Reporting Summary linked to this article.

### Supplementary information

Supplementary Information

Description of Additional Supplementary Files

Reporting Summary

### Source data

Source Data

## Data Availability

All data supporting the findings of this study are provided within the paper and in its Supplementary dataset/Source data.xlsx files. The uncropped Western blots are also provided in the Source data files. Source data are provided with this paper.
