# Supplementary material for: FSH and ApoE4 contribute to Alzheimer’s disease-like pathogenesis via C/EBPβ/δ-secretase in female mice
Source: Nat Commun. 2023 Oct 18;14:6577. doi: 10.1038/s41467-023-42282-7 (PMC10584868; doi:10.1038/s41467-023-42282-7)
Supplement: Supplementary file 1 — Supplementary Information [file 41467_2023_42282_MOESM1_ESM.pdf]

Supplementary Figure 1

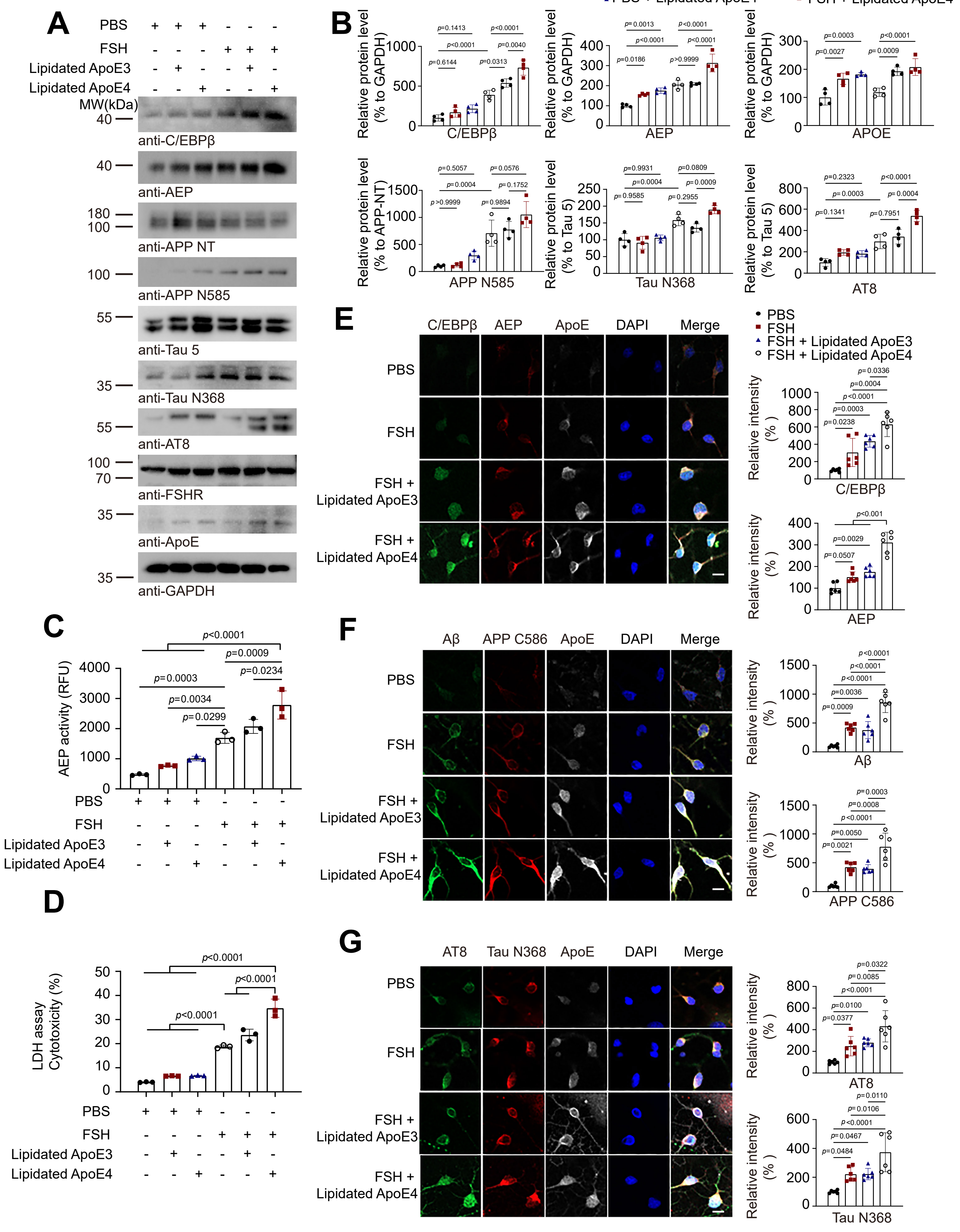

Supplementary Figure 1. FSH and lipidated ApoE4 additively activated C/EBPβ/δ-secretase pathway.

Primary neurons (DIV. 13) were treated with vehicle, FSH (30 ng/ml), or lipidated ApoE3 or ApoE4 for 48 hours. (A) Representative images of Western blot showed that FSH and lipidated ApoE4 additively increased the expression of C/EBPβ, active AEP, APP, Tau and their cleavage forms (APP N585, Tau N368). (B) Quantification of Western blot data (mean ± SEM, n = 4 independent experiments, One-way ANOVA test, followed by Tukey’s multiple comparisons test). (C) AEP enzymatic activity assay showed FSH and lipidated ApoE4 additively increased AEP enzymatic activities. Data were shown as mean ± SEM of 3 independent experiments (One-way ANOVA test, followed by Tukey’s multiple comparisons test). (D) LDH cytotoxicity assay showed FSH and lipidated ApoE4 treatment increased the cytotoxicity. Data represent mean ± SEM of 3 independent experiments (One-way ANOVA test, followed by Tukey’s multiple comparisons test). (E-G) Immunofluorescent staining showed the effect of FSH (30 ng/ml) on C/EBPβ (red) /AEP (green), Aβ (red) /APP C586 (green), AT8 (red) /Tau N368 (green) accumulation in the primary rat cortical neuron cultured with lipidated ApoE3/4 proteins (Scale bar, 10 μm). Lipidated-ApoE4 increased the stimulatory actions of FSH on C/EBPβ/AEP pathway. Data were shown as mean ± SEM (n = 6 slices from 3 independent experiments, One-way ANOVA test).

Supplementary Figure 2

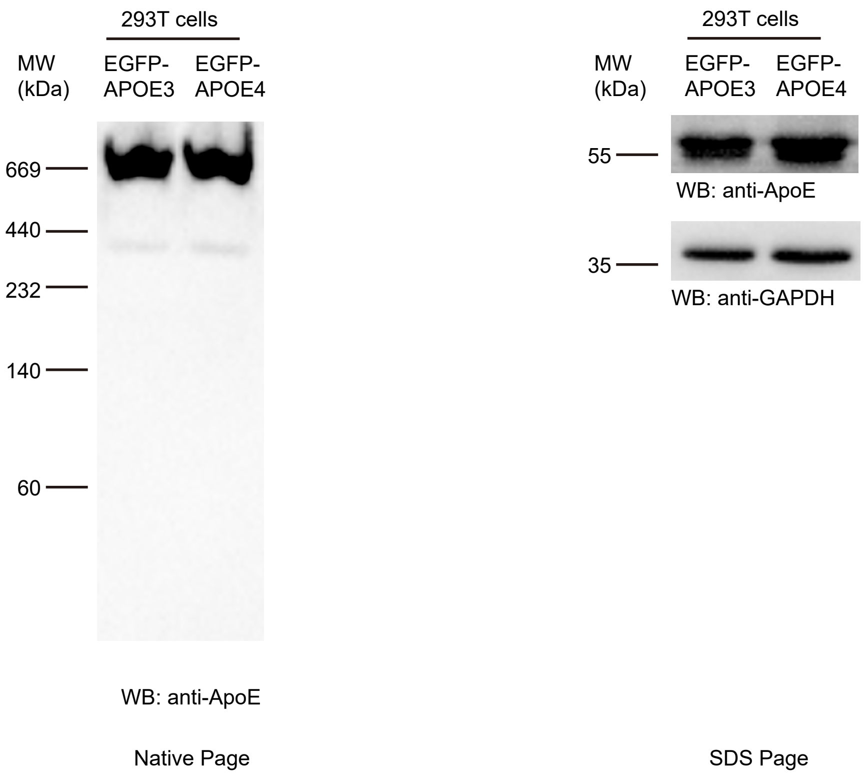

Supplementary Figure 2. Lipidated ApoE from HEK293T cells.

Conditioned medium from transfected HEK293T cells was isolated and analyzed by (A) native gel and western blot. By native gel, ApoE3 or ApoE4 proteins shows the expected high molecular weight smeared band which suggests it is properly lipidated (A). By Western blot, ApoE3 and ApoE4 showed similar purity in the conditioned medium separately (B).

Supplementary Figure 3

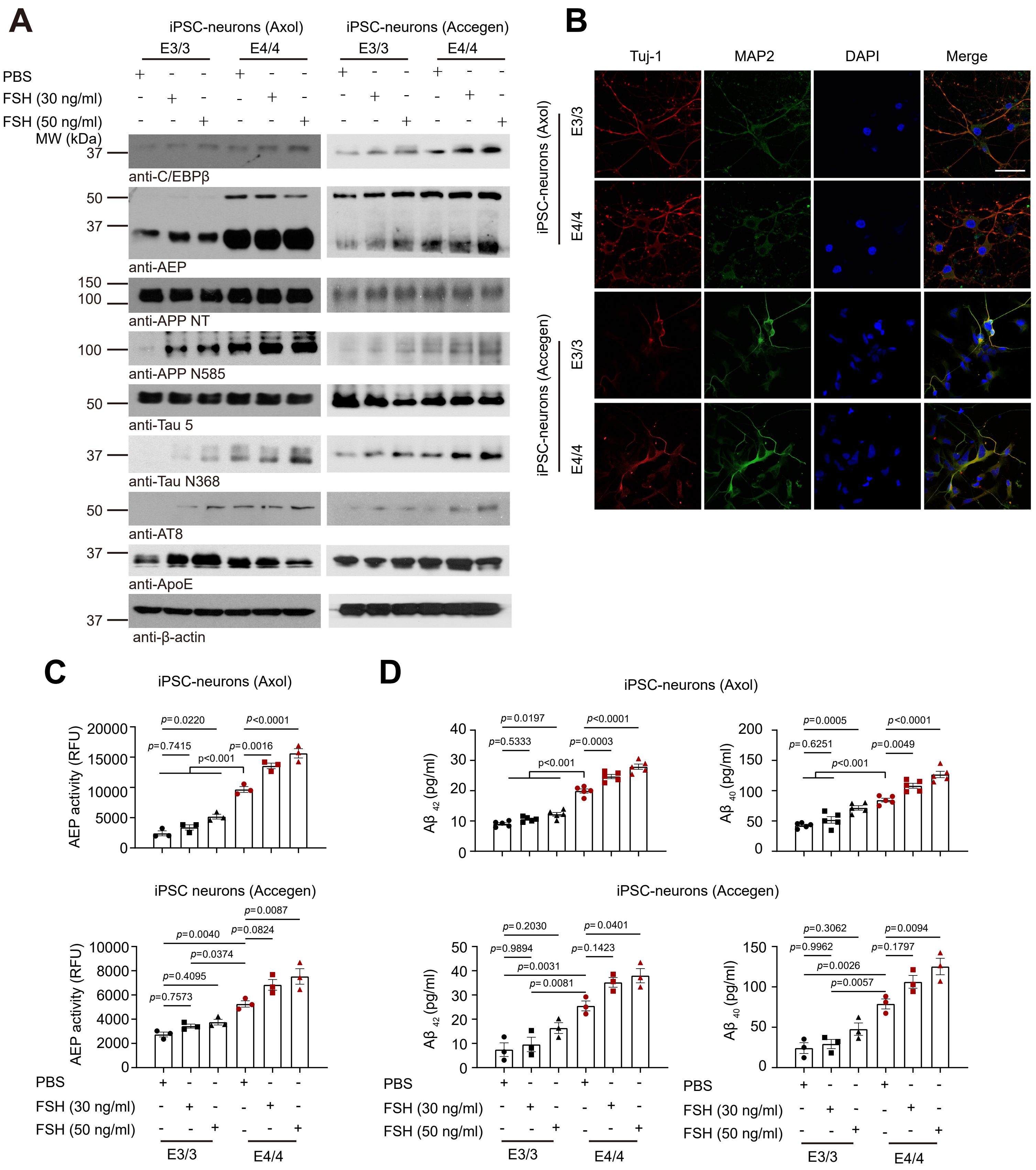

Supplementary Figure 3. FSH significantly activates C/EBPβ/δ-secretase pathway in human ApoE4/4 iPSC Neurons.

Both ApoE3/3 and ApoE4/4 AD patients' iPSC cells (two different strains) were differentiated into mature neurons, then treated with vehicle or FSH (30 ng or 50 ng) for 48 hours, respectively. (A) Western blot showed FSH treatment increased the protein expression of C/EBPβ, active AEP, APP, Tau and their cleavage forms (APP N585, Tau N368) in a dose-dependent manner, especially in ApoE4/4 group. (B) IF staining of differentiated human AD mature neurons from iPSC cells, validated with anti-MAP2 (green) and anti-Tuj1 (red) antibody (scale bar, 50 μm). (C) AEP enzymatic activity was quantitatively assessed. Data are shown as mean ± SEM of 3 independent experiments (One-way ANOVA test, followed by Tukey's multiple comparisons test). (D) Aβ40 and Aβ42 levels in the medium measured by ELISA assay. Data are shown as mean ± SEM. (n = 3 or 5 independent experiments, One-way ANOVA test, followed by Tukey's multiple comparisons test).

Supplementary Figure 4

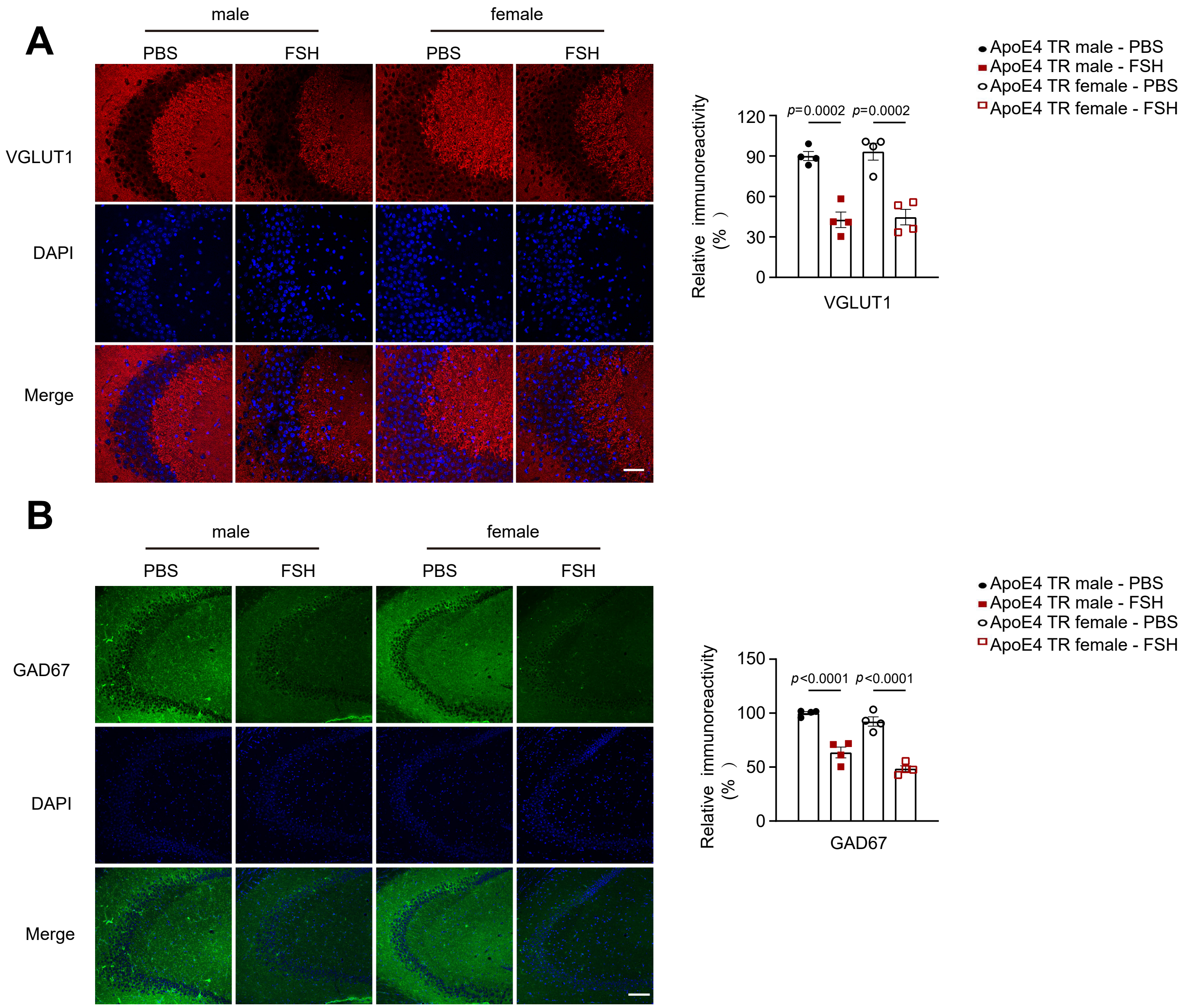

Supplementary Figure 4. FSH treatment decreased VGLUT1 and GAD67 expression both in male and female ApoE4-TR mice.

(A) IF staining showed decreased VGLUT1 (red) expression in the hippocampal CA3 region in FSH-treated male and female ApoE4-TR mice (scale bar, 50 μm). Quantification of VGLUT1 immunoreactivity. Data represent mean ± SEM (n = 4 mice per mice, One-way ANOVA test, followed by Tukey's multiple comparisons test). (B) IF staining showed decreased GAD67 (green) expression in the hippocampal CA3 region in FSH-treated male and female ApoE4-TR mice (scale bar, 100 μm). Quantification of GAD67 immunoreactivity. Data represent mean ± SEM (n = 4 mice per mice, One-way ANOVA test, followed by Tukey's multiple comparisons test).

Supplementary Figure 5

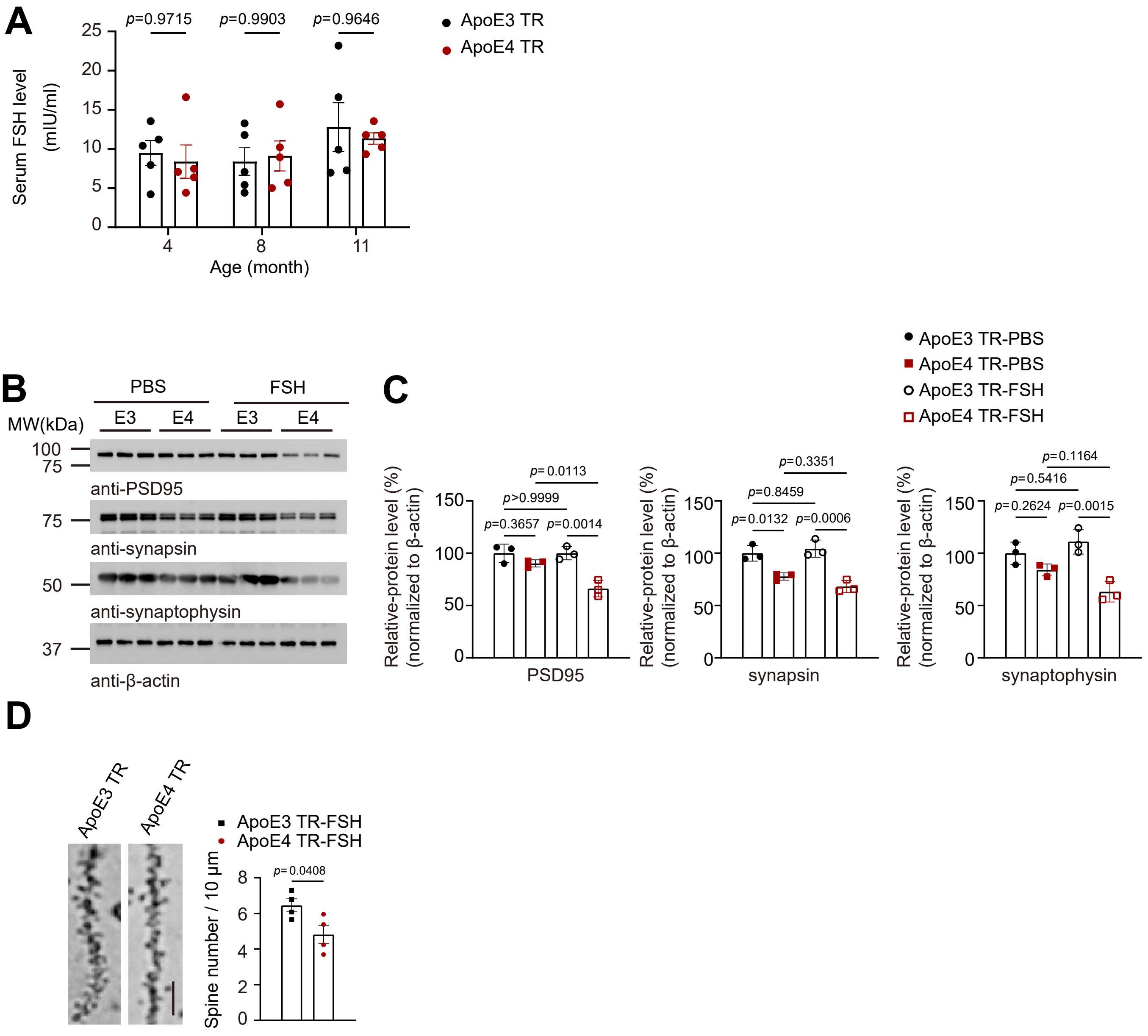

Supplementary Figure 5. FSH treatment decreased synapses in ApoE4-TR mice not ApoE3-TR mice.

(A) Serum FSH levels of female ApoE3-TR or ApoE4-TR mice at different ages (n = 5 mice each group, two-way ANOVA). (B) Western blot showed FSH treatment decreased PSD 95, synapsin and synaptophysin, triggering severe synapse loss in female ApoE4-TR mice. (C) Quantification of protein expression. Data represent mean ± SEM (n = 3 mice per group, One-way ANOVA test, followed by Tukey's multiple comparisons test). (D) Golgi staining of the brain sections from the CA1 region of the hippocampus showed reduced spine numbers in FSH treated ApoE4-TR mice compared ApoE3-TR mice (scale bar, 10 μm). Quantification of the dendritic spine density, which was calculated as the number of dendritic branches per 10 μm dendrites. Data were shown as mean ± SEM (n = 4 mice per group, unpaired t test with Welch's correction).

Supplementary Figure 6

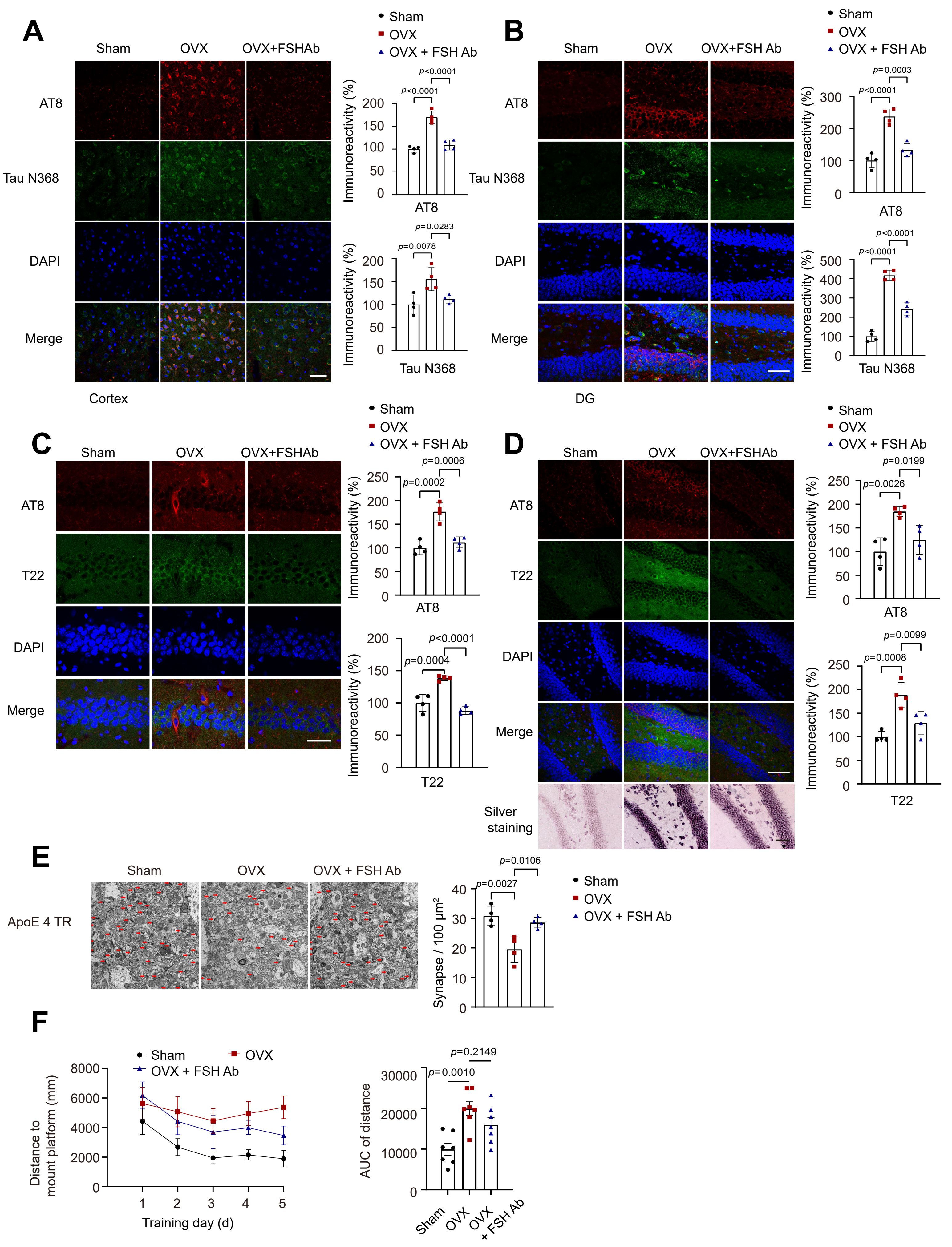

Supplementary Figure 6. FSH antibody treatment alleviated OVX-induced Tau phosphorylation and Tau aggregation.

(A&B) IF staining showed FSH antibody treatment suppressed OVX-induced AT8 (red) and Tau N368 (green) expression in the cortex (A) and the hippocampal DG region (B) of ApoE4-TR mice (scale bar, 50 μm). Quantification data were shown as mean ± SEM (n = 4 mice per group, One-way ANOVA test, followed by Tukey's multiple comparisons test). (C&D) IF staining showed FSH antibody treatment lessened OVX-induced AT8 (red) and T22 (green) expression in the hippocampal CA1 (C) and DG regions (D) in ApoE4-TR mice (scale bar, 50 μm). Quantification data were presented as mean ± SEM (n = 4 mice per group, One-way ANOVA test, followed by Tukey's multiple comparisons test). Sliver Staining of the hippocampus DG region(D, lower panels) (scale bar, 50 μm). (E) Electron microscopy analysis of the synapses. Arrows indicated the synapses (scale bar, 1 μm). Data represent mean ± SEM (n = 4 mice per group, One-way ANOVA test, followed by Tukey's multiple comparisons test). (F) Morris water maze analysis of cognitive functions. FSH antibody treatment attenuated OVX-induced learning and memory impairments in female ApoE4-TR mice. Data were shown as mean ± SEM (n = 7 mice per group, two-way ANOVA for distance, One-way ANOVA for AUC distance).

Supplymentary Figure 7

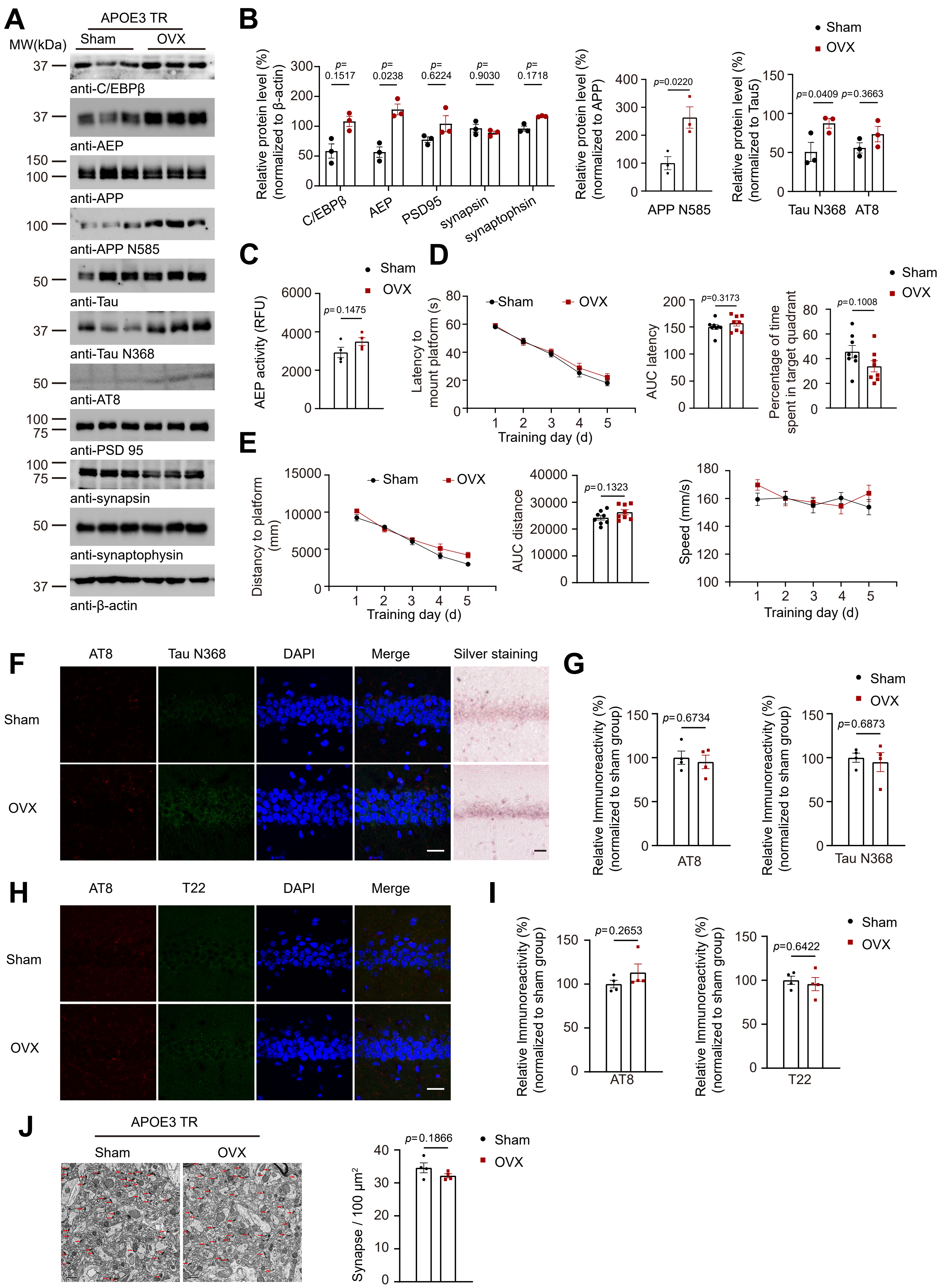

Supplementary Figure 7. Ovariectomy partially triggers C/EBPβ/AEP pathway activation, but not induced AD pathology in female ApoE3-TR mice.

Five months old female ApoE3 TR mice were subjected to sham or ovariectomy (OVX). Morris water maze were performed 8 weeks after OVX, and then the mice were sacrificed. (A) Representative images of Western blot analysis showed increased C/EBPβ, AEP, cleaved APP, p-Tau expression in APOE3-TR mouse brains after OVX. (B) Quantification of protein expression (mean ± SEM, n = 3 mice per group, two-way ANOVA or One-way ANOVA followed by Tukey's multiple comparisons test). (C) AEP enzymatic activities were increased after OVX, which was reduced upon FSH antibody treatment. Data were shown as mean ± SEM (n = 4 mice per group, unpaired t test with Welch's correction). (D&E) Morris water maze analysis of cognitive functions showed there's no learning and memory impairments in ApoE3-TR mice after OVX. Data were shown as mean ± SEM (n = 8 mice per group, two-way ANOVA for latency, distance and speed analyze, unpaired t test with Welch's correction for AUC latency, percentage of time in target quadrant and AUC distance). (F-I) AT8 (red)/Tau N368 (green) (F) and AT8 (red)/T22 (green) (H) Immunoreactivity in the hippocampus of ApoE3-TR mice after OVX were detected by Immunofluorescent (IF) staining (scale bar, 50 μm). Silver Staining of the hippocampus CA1 region (F, the right panels) showed no obvious proteinaceous deposits in ApoE3-TR mice after OVX (scale bar, 50 μm). Quantification of AT8/Tau N368 (G) and AT8/T22 (I) Immunoreactivity. Data were presented as mean ± SEM (n = 4 mice per group, unpaired t test with Welch's correction). (J) Electron microscopy analysis of the synapses (scale bar, 1 μm). Arrows indicated the synapses. Data were presented as mean ± SEM (n = 4 mice per group, unpaired t test with Welch's correction).

Supplementary Figure 8

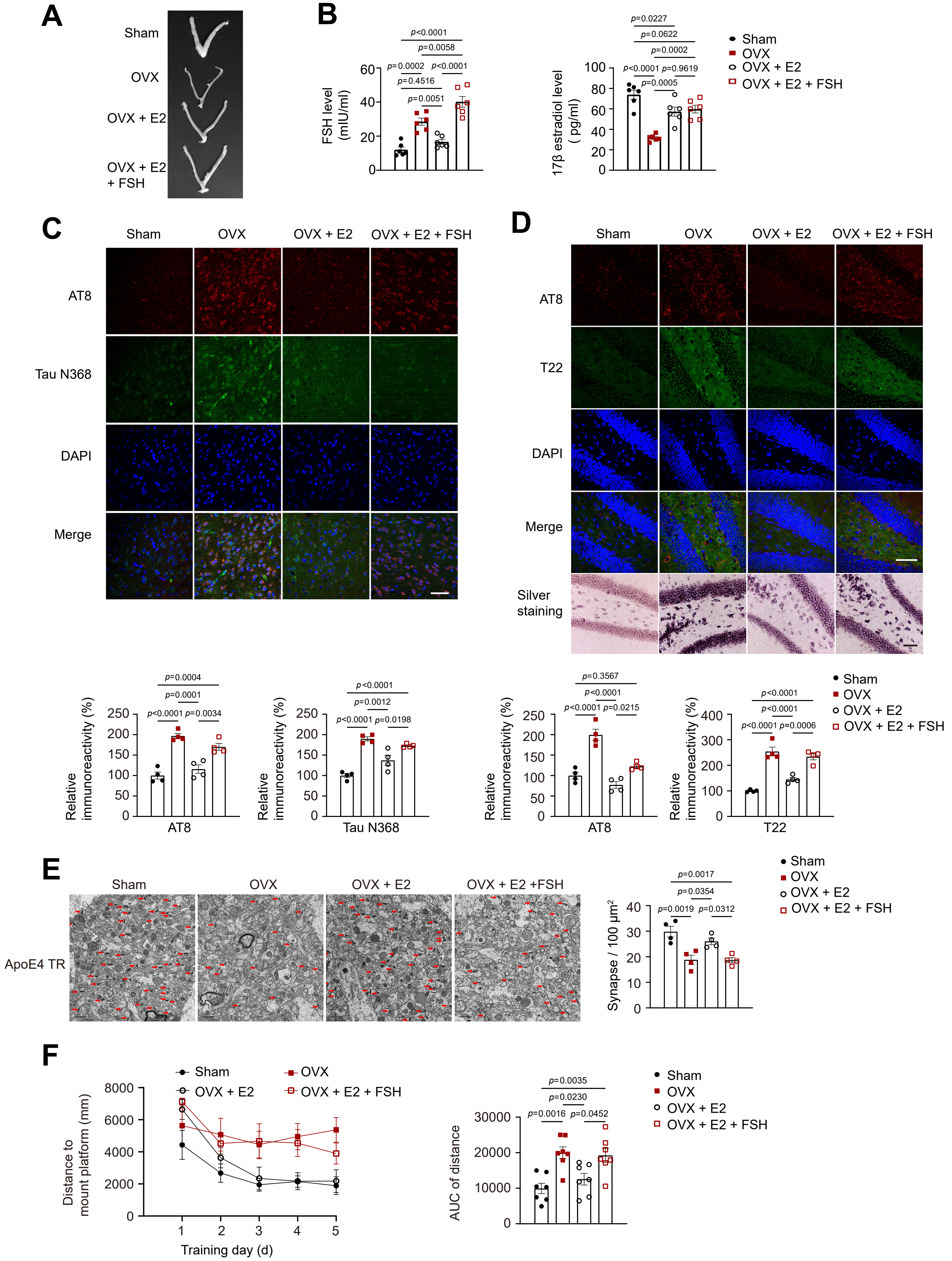

Supplementary Figure 8. FSH elevation but not estrogen deficiency is responsible for OVX-induced AD pathologies in ApoE4-TR mice.

(A) Morphology of the uterus. Ovariectomized ApoE4 TR mice displayed hypoplastic thread-like uteri, the E2 supplement partially suppressed OVX-induced uterus atrophy. (B) Serum levels of FSH and 17 β-estradiol. Data were shown as mean ± SEM (n = 6 mice per group, One-way ANOVA test, followed by Tukey's multiple comparisons test). (C) IF staining showed increased AT8 (red) and Tau N368 (green) expression in the cortex of OVX and OVX+E2+FSH group (scale bar, 50 μm). Data were presented as mean ± SEM (n = 4 mice per group, One-way ANOVA test, followed by Tukey's multiple comparisons test). (D) IF staining showed increased AT8 (red) and T22 (green) expression in the hippocampal DG region of OVX and OVX+E2+FSH group (scale bar, 50 μm). Data represent mean ± SEM (n = 4 mice per group, One-way ANOVA test, followed by Tukey's multiple comparisons test). Silver staining (the lower panels) showed increased proteinaceous deposits in OVX+E_2+FSH mice (scale bar, 50 μm). (E) Electron microscopy analysis of the synapses (scale bar, 1 μm). Arrows indicated the synapses. Data represent mean ± SEM (n = 4 mice per group, One-way ANOVA test, followed by Tukey's multiple comparisons test). (F) Morris water maze analysis of cognitive functions. Data were shown as mean ± SEM (n = 7 mice per group, two-way ANOVA for distance, One-way ANOVA for AUC distance).

Supplementary Figure 9

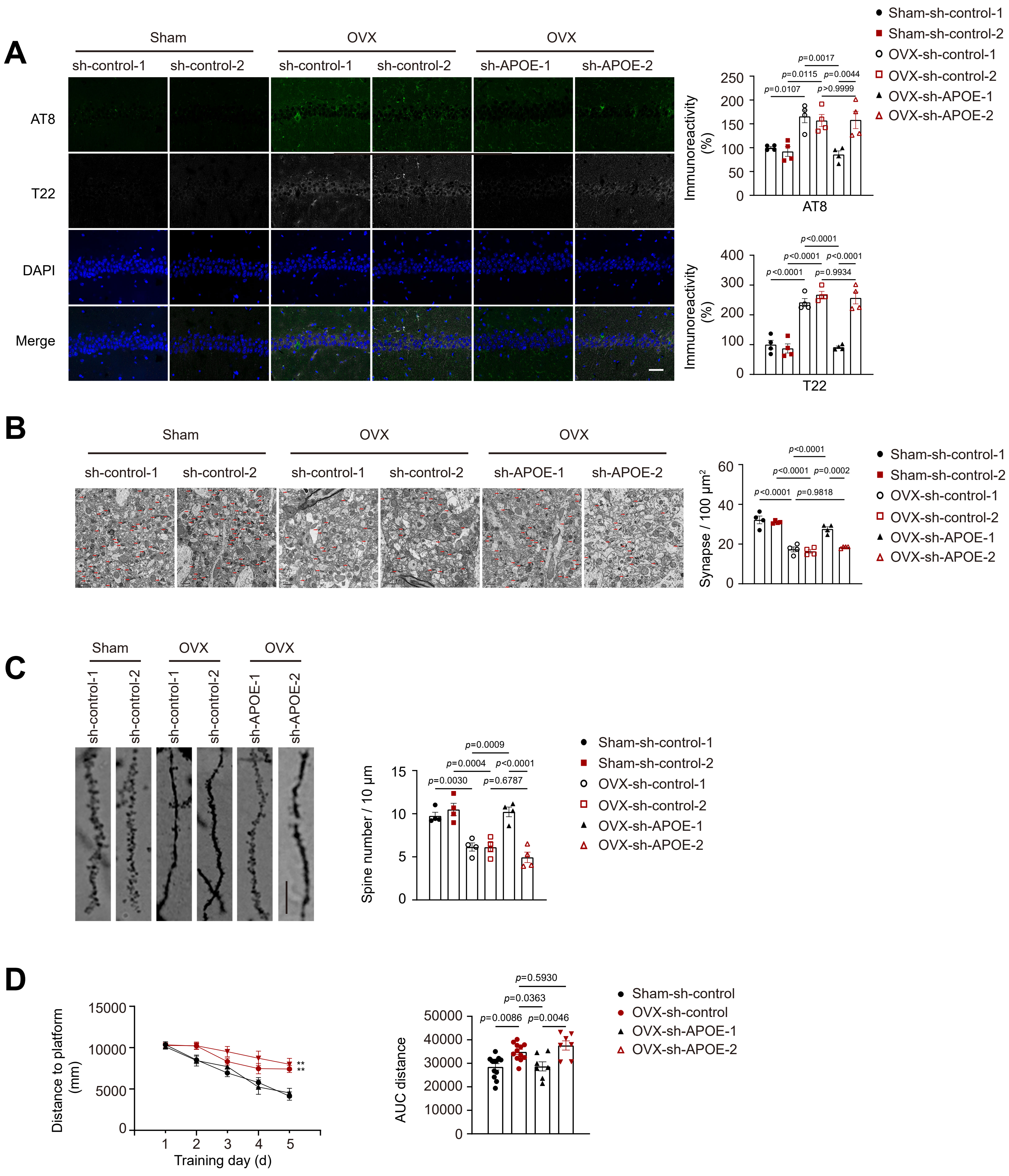

Supplementary Figure 9. Neuronal ApoE4 synergy with FSH promotes AD pathology.

(A) IF staining showed AT8 (red) and T22 (gray) in the hippocampus (left panels, scale bar, 50 μm). Quantification of AT8 and T22 immunoreactivity (right panels). Data were shown as mean ± SEM (n = 4 mice per group, One-way ANOVA followed by Tukey's multiple comparisons test). (B) Electron microscopy analysis of the synapses (scale bar, 1 μm). Arrows indicated the synapses. Data represent mean ± SEM (n = 4 mice per group, One-way ANOVA test, followed by Tukey's multiple comparisons test). (C) Golgi staining of the brain sections from the CA1 region of the hippocampus (scale bar, 10 μm). Quantification of the dendritic spine density, which was calculated as the number of dendritic branches per 10 μm dendrites. Data were presented as mean ± SEM (n = 4 mice per group, One-way ANOVA followed by Tukey's multiple comparisons test). (D) Morris water maze analysis of cognitive functions. Data were shown as mean ± SEM (n=7 or 12 mice per group, two-way ANOVA for distance, One-way ANOVA for AUC distance).
