# Supplementary material for: FSH and ApoE4 contribute to Alzheimer’s disease-like pathogenesis via C/EBPβ/δ-secretase in female mice
Source: Nat Commun. 2023 Oct 18;14:6577. doi: 10.1038/s41467-023-42282-7 (PMC10584868; doi:10.1038/s41467-023-42282-7)
Supplement: Supplementary file 2 — Description of Additional Supplementary Files [file 41467_2023_42282_MOESM2_ESM.pdf]

Supplementary data legends

File name: Supplementary Figures.PDF
Description: Supplementary Figure 1-9 and its figure legends.

File name: Source data.xlsx
Description: Source data.xlsx contains all the raw data for each figure.
