# Supplementary material for: FSH and ApoE4 contribute to Alzheimer’s disease-like pathogenesis via C/EBPβ/δ-secretase in female mice
Source: Nat Commun. 2023 Oct 18;14:6577. doi: 10.1038/s41467-023-42282-7 (PMC10584868; doi:10.1038/s41467-023-42282-7)
Supplement: Supplementary file 3 — Reporting Summary [file 41467_2023_42282_MOESM3_ESM.pdf]

nature portfolio

Corresponding author(s): Keqiang Ye

Last updated by author(s): Aug 16, 2023

Reporting Summary

Nature Portfolio wishes to improve the reproducibility of the work that we publish. This form provides structure for consistency and transparency in reporting. For further information on Nature Portfolio policies, see our Editorial Policies and the Editorial Policy Checklist.

Statistics

For all statistical analyses, confirm that the following items are present in the figure legend, table legend, main text, or Methods section.

n/a	Confirmed	
☐	☒	The exact sample size (*n*) for each experimental group/condition, given as a discrete number and unit of measurement
☐	☒	A statement on whether measurements were taken from distinct samples or whether the same sample was measured repeatedly
☐	☒	The statistical test(s) used AND whether they are one- or two-sided<br>*Only common tests should be described solely by name; describe more complex techniques in the Methods section.*
☒	☐	A description of all covariates tested
☐	☒	A description of any assumptions or corrections, such as tests of normality and adjustment for multiple comparisons
☐	☒	A full description of the statistical parameters including central tendency (e.g. means) or other basic estimates (e.g. regression coefficient) AND variation (e.g. standard deviation) or associated estimates of uncertainty (e.g. confidence intervals)
☐	☒	For null hypothesis testing, the test statistic (e.g. *F*, *t*, *r*) with confidence intervals, effect sizes, degrees of freedom and *P* value noted<br>*Give P values as exact values whenever suitable.*
☒	☐	For Bayesian analysis, information on the choice of priors and Markov chain Monte Carlo settings
☒	☐	For hierarchical and complex designs, identification of the appropriate level for tests and full reporting of outcomes
☒	☐	Estimates of effect sizes (e.g. Cohen's *d*, Pearson's *r*), indicating how they were calculated

Our web collection on statistics for biologists contains articles on many of the points above.

Software and code

Policy information about availability of computer code

Data collection	For Morris Water Maze tests, the data were captured and analyzed by MazeScan (Clever Sys, Inc.) .
Data analysis	ImageJ version: 1.53a (Bethesda, Maryland, USA) was used or analysis of immunofluorescent and immunoblots images. Graphpad Prism v.9.1.0 was used to analyze the data.

For manuscripts utilizing custom algorithms or software that are central to the research but not yet described in published literature, software must be made available to editors and reviewers. We strongly encourage code deposition in a community repository (e.g. GitHub). See the Nature Portfolio guidelines for submitting code & software for further information.

Data

Policy information about availability of data

All manuscripts must include a data availability statement. This statement should provide the following information, where applicable:

- Accession codes, unique identifiers, or web links for publicly available datasets
- A description of any restrictions on data availability
- For clinical datasets or third party data, please ensure that the statement adheres to our policy

All data associated with this study are present in the paper or the Supplementary information. Sourse data, including Excel spreadsheets and Western blots, will be deposited on the Nature Communications website. There are no restrictions on data availability.

Human research participants

Policy information about studies involving human research participants and Sex and Gender in Research.

Reporting on sex and gender	The used iPSC-derived NSCs were obtained from two donors: ax0111 from AD patient with ApoE4/4 genotype, ax0112 from AD patient with ApoE3/3 genotype (Axol Bioscience, Cambridge, UK). Another lines of human iPSC used in the experiment is from Accegen Biotechnology, USA. The gender of all the donors is female.
Population characteristics	ax0111 from AD patient with ApoE4/4 genotype is a 87 years old female.<br>ax0112 from AD patient with ApoE3/3 genotype 38 years old female.<br>The other two donor information of the iPSC cells from Accegen Biotechnology is not provided by the company.
Recruitment	The iPSC cell lines used in this experiment are commercial one,which are purchased from Axol Bioscience and Accegen Biotechnology. They don't provide the information on the product instruction.
Ethics oversight	The iPSC cell lines used in this experiment are commercial one,which are purchased from Axol Bioscience and Accegen Biotechnology. They don't provide the information on the product instruction.

Note that full information on the approval of the study protocol must also be provided in the manuscript.

Field-specific reporting

Please select the one below that is the best fit for your research. If you are not sure, read the appropriate sections before making your selection.

[x] Life sciences   [ ] Behavioural & social sciences   [ ] Ecological, evolutionary & environmental sciences

For a reference copy of the document with all sections, see nature.com/documents/nr-reporting-summary-flat.pdf

Life sciences study design

All studies must disclose on these points even when the disclosure is negative.

Sample size	No statistical methods were used to calculate sample size. Instead, sample sizes were determined on the basis of previous studies by Dr. Ye's and other groups (Nature, 2022)[PMID: 35236988], (Molecular Neurodegeneration, 2022)[PMID: 35093145], (EMBO J, 2021) [PMID: 34260075) .
Data exclusions	No data were excluded.
Replication	For each in vitro experiment, the data were obtained from at least 3 experimental replicates. In vivo experiment, the data were collected from at least 3 mice (biological replicates). The detail information is in the figure legend.
Randomization	Mice were randomly selected to be assigned into different group.
Blinding	Behavioral studies were conducted in the Rodent Behavioral Core by technicians who were unaware of the mouse groups. Data collection and analyses were automated for objective measurements, namely ELISA and AEP enzymatic activity. Findings were consistent between the automated objective measures and more subjective measurements, such as immunohistochemistry, histology and Western blotting, within the same experiment.

Reporting for specific materials, systems and methods

We require information from authors about some types of materials, experimental systems and methods used in many studies. Here, indicate whether each material, system or method listed is relevant to your study. If you are not sure if a list item applies to your research, read the appropriate section before selecting a response.

Materials & experimental systems

n/a	Involved in the study	
[ ]	[x]	Antibodies
[ ]	[x]	Eukaryotic cell lines
[x]	[ ]	Palaeontology and archaeology
[ ]	[x]	Animals and other organisms
[x]	[ ]	Clinical data
[x]	[ ]	Dual use research of concern

Methods

n/a	Involved in the study	
[x]	[ ]	ChIP-seq
[x]	[ ]	Flow cytometry
[x]	[ ]	MRI-based neuroimaging

Antibodies

Antibodies used	Antibody to C/EBPβ (HT-7) (catalog#: sc-7962, 1:1000 dilution for western blotting and 1:200 for immunofluorescence) was from Santa Cruz; anti-AEP (6E3) was a gift from Dr. Colin Watts (1:1000 dilution for western blotting), Professor of Immunobiology, Division of Cell Signaling and Immunology, College of Life Sciences, University of Dundee, Dundee, UK; antibodies to phosphor-Tau (Ser202-Thr205) (AT8, catalog#: MN1020, 1:1000 dilution for western blotting and 1:300 for immunofluorescence), phosphor-Tau (Thr212, Ser214) (AT100, catalog#: MN1020, 1:200 for immunohistochemistry) and IBA1 (catalog#: PA5-18039, 1:500 dilution for immunofluorescence) were from Thermo Fisher Scientific; antibodies to AEP (D6S4H) (catalog#: 93627, 1:2000 dilution for western blotting and 1:400 for immunofluorescence), PSD95 (catalog#: 2507, 1:1000 dilution for western blotting) and synapsin (catalog#: 5297, 1:1000 dilution for western blotting)were purchased from Cell Signaling Technology; antibody to Tau 5 (catalog#: MAB361, 1:2000 dilution for western blotting), β-actin (catalog#: A5316, 1:3000 dilution for western blotting) and GFAP (catalog#: MAB360, 1:400 dilution for immunofluorescence)were from Sigma-Aldrich; antibody to Aβ (4G8) (catalog#: 800701, 1:200 dilution for immunofluorescence) was obtained from Biolegend; Antibody to ApoE (catalog#: AB947, 1:1000 dilution for western blotting), T22 (catalog#: ABN454, 1:600 dilution for immunofluorescence) and GAD67 (catalog#: MAB5406, 1:2000 dilution for western blotting and 1:500 dilution for immunofluorescence) was bought from Millipore Sigma; Antibody to VGLUT1 (catalog#: 135311, 1:500 dilution for immunofluorescence) were from Synaptic Systems; antibody to synaptophysin (catalog#: ab32127, 1:1000 dilution for western blotting) were from Abcam; antibodies to Tau N368 (1:3000 dilution for western blotting and 1:700 for immunofluorescence), APP N585 and APP N373 ((1:1000 dilution for western blotting) were developed in the Ye lab. The anti-FSHβ polyclonal antibody (FSH Ab) was developed and characterized in the Zaidi lab. Human Aβ40 (catalog#: KHB3481), Aβ42 (catalog#: KHB3544), mouse Aβ40 (catalog#: KMB3481) and mouse Aβ42 (catalog#: KMB 3441) were purchased from Invitrogen. sta. The AEP substrate Z-Ala-Ala-Asn-AMC (catalog#: 4033201) was from Bachem. Recombinant human FSH used in vitro and in vivo experiment was from Sigma-Aldrich (Catalog#: F4021) and EastCoast Bio (Catalog#: LA252), respectively. 90–day–release pellets containing 0.36 mg 17β-estradiol (catalog#: NE121) were purchased from Innovative Research of America. The AAV9-syn-sh-APOE virus, AAV5-gfabc1d-sh-APOE virus, AAV9-syn-sh-control virus and AAV5-gfabc1d-sh-control virus (2 × 1012 vector genomes per ml) were obtained from BrainVTA Co., Ltd. (Wuhan, China). All chemicals not mentioned above were purchased from Sigma-Aldrich.
Validation	(1) Antibody to C/EBPβ (H-7) detects C/EBPβ of mouse, rat and human origin by Western blotting, immunoprecipitation, immunofluorescence and immunohistochemistry (PMID: 29725016).<br>(2) Anti-AEP (6E3) detects mouse and human LGMN by western blotting, immunoprecipitation, immunofluorescence and immunohistochemistry (PMID: 12860980, 35236988)<br>(3) Antibody to AEP(D6S4H) (catalog#: 93627, clone D6S4H) interacts with human, mouse and rat proteins on immunofluorescence and Western blotting (PMID: 31793911).<br>(4) Antibody to pTauS202,T205 (AT8, catalog#: MN1020) targets PHF-tau (Ser202/Thr205) and phosphor-Tau (Thr212, Ser214) (AT100, catalog#: MN1020) targets PHF-tau (Thr212, Ser214) has been validated in ELISA, immunofluorescence, immunohistochemisty, and Western blot applications and shows reactivity with human, mouse, rat, chicken, non-human primate and Hamster samples (PMID: 12860980, 35236988, 33285637).<br>(5) Antibody to Tau (210-241, catalog#: MAB361, clone tau-5) detects protein from rat, human and cow and is used for Western blotting. (PMID: 25471585, 33285637,28826672, 2532680)<br>(6) Antibody to Aβ (atalog#: 800701, clone 4G8) is reactive to human and mouse β amyloid (amino acid residues 17-24), as well as detects precursor forms, and is used for immunohistochemistry and immunofluorescence in human, mouse. (PMID: 35236988, 33285637)<br>(7) Antibody to GFAP (mouse monoclonal, catalog# MAB360, clone GA5) has been validated for immunofluorescence, immunohistochemisty, and Western blot of pig, rat, mouse and human GFAP protein in more than 65 citations. (PMID: 29297157)<br>(8) Antibody to IBA1 (catalog# PA5-18039) has been validated for use in WB, IHC, IHC-P/FFPE of human, mouse, rat. (PMID: 33716161).<br>(9) Antibody to ApoE (catalog#: AB947) is reactive to human, Primate ApoE, and used for Immunohistochemistry and Western Blotting (PMID: 33716161, 33895869)<br>(10) Antibody to VGLUT1 (catalog#: 135311) has been validated for immunofluorescence, immunohistochemisty, Elisa, immunoprecipitation and Western blot in rat, mouse sample (PMID: 34502320, 21435559).<br>(11) Antibody to GAD67 (catalog#: MAB6406) has been validated for immunofluorescence, immunohistochemisty and Western blot in huamn, rat, mouse sample (PMID: 26042808, 24723034)<br>(12) Antibody to T22 (catalog#: ABN454) has been validated for immunofluorescence, immunohistochemisty, Elisa, immunoprecipitation and Western blot in human, mouse sample (PMID: 22253473, 23632019)<br>(13) Antibody to β -actin (catalog#: A5316, clone AC-74) detects protein on Western blotting, immunofluorescence and immunohistochemistry in tissues from humans, rat, mouse, pig, dog, sheep and guinea pig.<br>(14) The polyclonal antibody to the Tau1-368, APP1-585 and APP1-373were generated in the rabbit and validated functionally in the Ye lab (Xiong et al. 2022, Nature, PMID: 35236988, Wang et al., 2017, Molecular Cell, PMID: 28826672, Zhang et al., 2014, Nat Med, PMID 2532680 and Zhang et al., 2015, Nat Comm, PMID: 26549211.).<br>(15) The polyclonal antibody to the 13-amino-acid-long FSHR-binding epitope of mouse FSH was generated in the goat and validated functionally in the Zaidi lab [Zhu et al., 2012, PNAS, PMID: 22908268 and Liu et al., Nature, 2017, PMID: 28538730].

Eukaryotic cell lines

Policy information about cell lines and Sex and Gender in Research

Cell line source(s)	The used iPSC-derived NSCs were obtained from two donors: ax0111 from AD patient with ApoE4/4 genotype, ax0112 from AD patient with ApoE3/3 genotype (Axol Bioscience, Cambridge, UK). Another lines of human iPSC used in the experiment is from Accegen Biotechnology, USA. All of the iPSC-derived NSCs and iPSC were commercial product.
Authentication	Not authenticated.

Mycoplasma contamination	Not tested for Mycoplasma.
Commonly misidentified lines (See ICLAC register)	None

Animals and other research organisms

Policy information about studies involving animals; ARRIVE guidelines recommended for reporting animal research, and Sex and Gender in Research

Laboratory animals	4 months old Female and male APOE4 Targeted Replacement (stock# 1549-F and 1549-M) and female APOE3 Targeted Replacement mice (stock# 1548-F) were obtained from Taconic Biosciences, Inc (United States).
Wild animals	None
Reporting on sex	For FSH i.p treatment group, we use both female and male APOE4 TR mice, female APOE3 TR mice. The female APOE4 TR mice were used in sham, ovariectomy(OVX), OVX+Fsh Ab, OVX+E2, OVX+E2+FSH group and AAV-sh-APOE+OVX group.
Field-collected samples	The study did not involve samples to be collected from the field.
Ethics oversight	The experiments were conducted according to the NIH animal care guidelines and Emory School of Medicine guidelines. The protocol was reviewed and approved by the Institutional Animal Care and Use Committee (IACUC) at Emory University.

Note that full information on the approval of the study protocol must also be provided in the manuscript.
